# Assessment of Prescription Analgesic Use in Older Adults With and Without Chronic Kidney Disease and Outcomes

**DOI:** 10.1001/jamanetworkopen.2020.16839

**Published:** 2020-09-30

**Authors:** Yun Han, Rajesh Balkrishnan, Richard A. Hirth, David W. Hutton, Kevin He, Diane E. Steffick, Rajiv Saran

**Affiliations:** 1 Division of Nephrology, Department of Internal Medicine, and the Kidney Epidemiology and Cost Center, University of Michigan, Ann Arbor; 2 University of Michigan School of Public Health, Department of Health Policy and Management, Ann Arbor; 3 University of Michigan School of Public Health, Department of Epidemiology, Ann Arbor

## Abstract

**Importance:**

Pain is a common symptom among patients with kidney disease. However, little is known about use of analgesics among patients aged 65 years or older with chronic kidney disease (CKD) who do not receive dialysis treatment.

**Objective:**

To assess national trends and geographic variations in use of opioids and prescription nonsteroidal anti-inflammatory drugs (NSAIDs) in older adults with and without CKD in the US (2006-2015) and examine associations between use of opioids and patient outcomes.

**Design, Setting, and Participants:**

This cohort study used the 5% Medicare claims data (2005-2015) to select 10 retrospective annual cohorts of Medicare Part D beneficiaries aged 65 years and older from 2006 to 2015 and a retrospective longitudinal cohort. Data were analyzed in August 2019.

**Exposures:**

CKD status and other comorbidities identified using *International Classification of Diseases, Ninth Revision, Clinical Modification* codes.

**Main Outcomes and Measures:**

Analgesic use was measured by overall use (proportion of ever used opioids/NSAIDs), long-term use (prescribed >90 days), and cumulative use (total annual days' supply). Patient outcomes included progression to end-stage kidney disease (ESKD) and all-cause mortality.

**Results:**

A total of 6 260 454 beneficiaries (9.6% identified with CKD by claims) were selected in the annual cohorts and 649 339 beneficiaries (8.3% identified with CKD) were selected in the longitudinal cohort. There was significant growth in opioid use (31.2%-42.4%) and NSAID use (10.7%-16.6%) among patients aged 65 years and older with CKD from 2006 to 2015. Long-term use of opioids increased during 2006 to 2014 (25.8%-36.7%) but decreased through 2015 at 35.6%, while long-term use of NSAIDs remained stable. Opioid use was higher in patients with CKD, particularly CKD stages 4 to 5 (odds ratio [OR], 1.35; 95% CI, 1.33-1.37; *P* < .001) compared with non-CKD. NSAID use was lower in patients with CKD stages 4 to 5 (OR, 0.55; 95% CI, 0.54-0.56; *P* < .001). Substantial geographic variations in analgesic use were observed across states (opioid use in CKD: 24.7%-54.3%; NSAID use in CKD: 11.2%-20.8%, 2012-2015). Opioid use was associated with progression to ESKD (hazard ratio [HR], 1.10; 95% CI, 1.04-1.16; *P* = .001) and death (HR, 1.19; 95% CI, 1.18-1.20; *P* < .001) independent of CKD status and other covariates. There was an inverse association between NSAID use and death (HR, 0.84; 95% CI, 0.83-0.85; *P* < .001).

**Conclusions and Relevance:**

Among Medicare patients with CKD, use of prescription analgesics, both opioid and NSAID, increased from 2006 to 2015. Optimizing pain management in a complex condition such as kidney disease should remain a priority for clinicians and researchers alike.

## Key Points

**Question:**

Is prescription analgesic use higher among Medicare patients with chronic kidney disease (CKD) compared with the general population?

**Findings:**

In this cohort study of more than 6 million Medicare Part D beneficiaries, use of both opioids and prescription nonsteroidal anti-inflammatory drugs (NSAIDs) increased substantially among patients with CKD aged 65 years or older from 2006 to 2015. Patients with CKD used significantly more opioids but less NSAIDs compared with the non-CKD population.

**Meaning:**

Results of this study suggest that optimization of pain management in CKD is warranted.

## Teaser

This cohort study assesses national trends and geographic variations in use of opioids and prescription nonsteroidal anti-inflammatory drugs (NSAIDs) in Medicare patients aged 65 years or older with and without chronic kidney disease in the US (2006-2015) and examines associations between use of opioids and patient outcomes.

## Introduction

Pain is one of most common symptoms experienced by patients with kidney disease.^1,2^ More than 70% of patients with chronic kidney disease (CKD) report experiencing pain.^3^ Pain has been associated with lower quality of life, high symptom burden, and greater risk of developing kidney disease, its progression, and mortality.^1,4^ Thus, management of pain is a vital component of comprehensive care for patients with CKD.^3^

The already high medication burden makes pain management even more complex in patients with CKD. There are no well-established guidelines for pain management specific to those individuals with kidney disease.^2,5,6,7,8^ Special precautions are needed to manage treatment of patients with reduced kidney function, who may be more prone to experiencing drug toxic effects, adverse effects, greater dose adjustment requirements, and drug interactions.^2^ Opioids and nonsteroidal anti-inflammatory drugs (NSAIDs) are the most commonly used analgesics. Opioids may have extended half-life in patients with advanced CKD, with substantial effects on the central nervous system, resulting in respiratory depression, hypotension, and addiction.^2,5,6^ Long-term opioid use is a risk factor for opioid abuse and dependence.^9^ Use of NSAIDs in patients with CKD may result in nephrotoxicity, fluid and electrolyte imbalances, hypertension, and other complications.^2,6,10,11,12,13^ Therefore, patients with CKD may be especially at risk of receiving suboptimal pain control and inappropriate use of prescription analgesics.^14^

The opioid epidemic has recently received considerable attention from clinicians, policy makers, and the public.^15^ However, to our knowledge, few studies have assessed use of analgesics and outcomes in patients with reduced kidney function, particularly with regard to the long-term effects.^3^ The present study sought first to examine trends from 2006 to 2015 in the use of opioids and prescription NSAIDs among older (age ≥65 years) patients with CKD in the US, and then to explore factors associated with opioid and NSAID use, as well as outcomes associated with their use, including development of end-stage kidney disease (ESKD) and death. We hypothesized that prescription analgesic use would be higher in patients with CKD compared with the general population. Furthermore, we postulated that use of prescription NSAIDs would be associated with more rapid kidney disease progression to ESKD and opioid use with both higher mortality and advanced-stage or progressive kidney disease.

## Methods

### Data Sources

This study used the 5% Medicare Part D claims data sample available to the United States Renal Data System (USRDS) (2005-2015). Data were linked to the USRDS ESKD database and therefore contained information pertaining to both ESKD and death. This study was performed under the USRDS Coordinating Center contract with the National Institutes of Health National Institute of Diabetes and Digestive and Kidney Diseases (NIDDK); research as part of the contract has been approved by the University of Michigan Institutional Review Board. Because data for the USRDS components are collected by federal mandate, there are no individual patient consent requirements. This study followed the Strengthening the Reporting of Observational Studies in Epidemiology (STROBE) reporting guideline for cohort studies. Data were analyzed in August 2019.

### Study Design

Ten annual cohorts of Medicare beneficiaries were identified to evaluate analgesic use among older adults with and without CKD. A visual depiction of the study design and sample selection is provided in eFigure 1 in the Supplement. Briefly, for each calendar year, patient demographic characteristics, CKD status, and comorbidities were assessed in the selection period, the year prior to the calendar year; and patients’ use of analgesics was assessed within the calendar year. Beneficiaries aged 65 years or older, continuously enrolled in fee-for-service Medicare and Part D, and with no diagnosis of ESKD in the selection period were selected. Presence of CKD and other comorbidities was defined as having at least 1 inpatient claim or 2 outpatient claims within the selection period with specified *International Classification of Diseases, Ninth Revision, Clinical Modification* (*ICD-9-CM*) codes.^16^ Investigated conditions and corresponding *ICD-9-CM* codes are summarized in eTable 1 in the Supplement. Patient CKD status, referring to whether the patient was diagnosed as having CKD and the stage of CKD, was determined from administrative claims with CKD-related *ICD-9-CM* codes and CKD stage-specific codes (585.x). Patients without CKD served as a control group in this study. The sample size of the annual CKD cohorts increased from 29 843 in 2006 to 93 755 in 2015.

A longitudinal cohort was developed to examine the association between analgesic prescription and patient outcomes. Study design and sample selection are depicted in eFigure 2 in the Supplement. Briefly, Part D enrollees in 2006-2015 were selected. The first observed date of Part D enrollment was the beginning of the 1-year selection period. Demographic characteristics, patient CKD status, comorbidities, and use of analgesics at baseline were assessed in the selection period using the same algorithm as the annual cohorts. Study participants were then followed after the end of the 1-year selection period. Medicare beneficiaries who were less than age 65 years at the index date, were enrolled in Medicare Advantage, or were without continuous prescription coverage in the 1-year selection period were excluded from this study. A total of 649 339 participants were selected.

### Measures

Analgesic use was assessed in the following 3 aspects: (1) overall use, as measured by the proportion of individuals with any prescription opioids/NSAIDs; (2) long-term use, measured by the proportion of use of prescription opioids/NSAIDs for greater than 90 days; and (3) cumulative use, measured by total annual days' supply of opioids/NSAIDs among users in a given calendar year. A list of National Drug Codes for opioids and NSAIDs was developed according to the American Hospital Formulary Society drug categories. Corresponding Medicare Part D claims were then extracted based on these National Drug Codes. Beneficiaries who had any opioid claim in the calendar year were classified as opioid users. Total days’ supply of opioids was calculated as the sum of all opioid prescriptions dispensed in the calendar year. A threshold of 91 days was applied to define chronic pain therapy.^10,17^ The measures were calculated in the same manner for prescription NSAID use.

Patient outcomes included progression to ESKD and all-cause mortality. The date of ESKD was defined as the initiation of kidney replacement therapy reported in the Medical Evidence form (Centers for Medicare & Medicaid Services [CMS] 2728). Death information was obtained from the ESKD Death Notification form (CMS 2746) for those who developed ESKD and from the Medicare 5% sample for those without onset of ESKD. Study participants were censored at date of death or end of the study period (December 31, 2015) when progression to ESKD was the event, whereas they were censored at the end date of this study when all-cause mortality was the event.

Demographic information was obtained from Medicare data sets. Investigated comorbidities included CKD-related conditions (hypertension, cardiovascular disease, diabetes) and pain related conditions (cancer, depression, back pain, neck pain, arthritis, headache, and HIV).^18^ Cardiovascular disease was defined as having of any of the following conditions: cerebrovascular accident, peripheral vascular disease, atherosclerotic heart disease, congestive heart failure, dysrhythmia, or other cardiac comorbidities.

### Statistical Analysis

Patient characteristics of the annual cohort (2006-2015) are summarized in eTable 2 in the Supplement. Characteristic differences between users and nonusers were assessed by *t* test and χ^2^ test (eTable 3 in the Supplement). Trends of opioid and prescription NSAID use by CKD status, race/ethnicity, comorbidities, and state were shown in line graphs. Generalized linear models were used for time-trend analyses (link function = logit, random component = binomial). These models were built considering repeated measures nested within study participants. Generalized linear models were further applied to explore patient factors associated with use of prescription opioids and NSAIDs. Geographic variation in use of opioids and prescription NSAIDs from 2006 to 2015 was assessed by CKD and presented in maps at state level. The 3 different measurements of analgesic use were aggregated at state level in 3 time periods: 2006-2008, 2009-2011, and 2012-2015. Changes in use of analgesics by the 3 time periods were presented by box plots (eFigures 6-11 in the Supplement).

In the outcome analyses, baseline characteristics of the longitudinal cohort were assessed by analgesic use using *t* test and χ^2^ test. Multivariate Cox proportional hazards regression models were applied to assess the association between analgesic use, development of ESKD, and all-cause mortality. The competing risk of death was taken into account when modeling development of ESKD. Statistical significance was set at 2-sided *P* < .05. All analyses were conducted using SAS version 9.4 (SAS Institute). Maps were developed using R software (R Project for Statistical Computing).

## Results

In this cohort study, a total of 6 260 454 beneficiaries (CKD 9.6%) were selected in the annual cohorts, and 649 339 beneficiaries (CKD 8.3%) were selected in the longitudinal cohort.

### Use of Analgesics

The proportion of Medicare Part D beneficiaries aged 65 years or older diagnosed with CKD increased from 5.4% in 2006 to 12.1% in 2015 (eTable 2 in the Supplement). A total of 6 260 454 beneficiaries were selected in the 10-year annual cohorts and 9.6% of them had received a diagnosis of CKD (601 718) (eTable 3 in the Supplement).

The CKD population was older (mean [SD] age, 79.2 [7.96] years vs 76.5 [7.7] years, with a greater proportion of male sex (43.3% vs 36.9%), Black race (11.73% vs 7.17%), and patients with comorbidities (eTable 3 in the Supplement). A total of 649 339 beneficiaries (prevalence of CKD, 8.3%) were selected in the longitudinal cohort. The mean (SD) age of the longitudinal cohort was 76.3 (7.8) years, 36.4% were male, and 82.7% were White (Table 1).

**Table 1.** Baseline Characteristics of the Longitudinal Cohort by Prescription Analgesics Use Status^a^

Characteristic	No. (%)				
	All (n = 649 339)	Opioids		Prescription NSAIDs	
		User (n = 210 145)	Nonuser (n = 439 194)	User (n = 141 498)	Nonuser (n = 507 841)
**Use of opioids**					
Ever used	210 145 (32.4)	210 145 (100)	0	71 280 (50.4)	138 865 (27.3)
Total annual days' supply, mean (SD)	28.6 (88.3)	88.3 (137.1)	0	46.9 (108.0)	23.5 (81.2)
Total annual days' supply categories					
0 d	439 204 (67.6)	0	439 194 (100)	70 220 (49.6)	368 984 (72.7)
1-90 d	152 817 (23.5)	152 817 (72.7)	0	50 520 (35.7)	102 297 (20.1)
91-180 d	20 435 (3.1)	20 435 (9.7)	0	7838 (5.5)	12 597 (2.5)
>180 d	36 883 (5.7)	36 883 (17.6)	0	12 920 (9.1)	23 963 (4.7)
**Use of NSAIDs**					
Ever used	507 841 (21.8)	138 865 (66.1)	368 976 (84.0)	141 498 (100)	0
Total annual days' supply, mean (SD)	25.8 (74.3)	41.7 (91.4)	18.1 (63.2)	118.2 (120.1)	0
Total annual days' supply categories					
0 d	507 858 (78.2)	138 871 (66.1)	368 987 (84.0)	0	507 841 (100)
1-90 d	86 166 (13.3)	41 719 (19.9)	44 447 (10.1)	86 166 (60.9)	0
91-180 d	20 638 (3.2)	11 083 (5.3)	9555 (2.2)	20 638 (14.6)	0
>180 d	34 677 (5.3)	18 472 (8.8)	16 205 (3.7)	34 677 (24.5)	0
**Demographic characteristics**					
Age, mean (SD)	76.3 (7.8)	76.5 (7.8)	76.2 (7.8)	75.6 (7.3)	76.5 (7.9)
Age group, y					
65 to <75	323 210 (49.8)	102 058 (48.6)	221 152 (50.4)	75 611 (53.4)	247 599 (48.8)
≥75 to <85	223 679 (34.4)	73 881 (35.2)	149 798 (34.1)	48 376 (34.2)	175 303 (34.5)
≥85	102 450 (15.8)	34 206 (16.3)	68 244 (15.5)	17 511 (12.4)	84 939 (16.7)
Male	236 617 (36.4)	67 363 (32.1)	169 254 (38.5)	44 407 (31.4)	192 210 (37.8)
Race/ethnicity					
White	536 921 (82.7)	176 532 (84.0)	360 389 (82.1)	109 406 (77.3)	427 515 (84.2)
Asian American	20 606 (3.2)	4523 (2.2)	16 083 (3.7)	6908 (4.9)	13 698 (2.7)
Other	12 773 (2.0)	3288 (1.6)	9485 (2.2)	3234 (2.3)	9539 (1.9)
Black	57 307 (8.8)	19 327 (9.2)	37 980 (8.6)	14 458 (10.2)	42 849 (8.4)
Unknown	2574 (0.4)	641 (0.3)	1933 (0.4)	663 (0.5)	1911 (0.4)
Hypertension	397 522 (61.2)	150 495 (71.6)	247 027 (56.2)	97 356 (68.8)	300 166 (59.1)
Cardiovascular disease	287 116 (44.2)	116 642 (55.5)	170 474 (38.8)	64 641 (45.7)	222 475 (43.8)
Diabetes	158 967 (24.5)	61 981 (29.5)	96 986 (22.1)	39 391 (27.8)	119 576 (23.5)
CKD status					
All CKD	53 788 (8.3)	25 910 (12.3)	27 878 (6.3)	11 009 (7.8)	42 779 (8.4)
Non-CKD	595 551 (91.7)	184 235 (87.7)	411 316 (93.7)	130 489 (92.2)	465 062 (91.6)
Stages 1-2	3348 (0.5)	1493 (0.7)	1855 (0.4)	767 (0.5)	2581 (0.5)
Stage 3	13 501 (2.1)	6116 (2.9)	7385 (1.7)	2580 (1.8)	10 921 (2.2)
Stages 4-5	7264 (1.1)	3520 (1.7)	3744 (0.9)	1112 (0.8)	6152 (1.2)
Unknown/other	29 675 (4.6)	14 781 (7.0)	14 894 (3.4)	6550 (4.6)	23 125 (4.6)
Cancer	62 804 (9.7)	27 456 (13.1)	35 348 (8.0)	13 159 (9.3)	49 645 (9.8)
Depression	39 745 (6.1)	20 796 (9.9)	18 949 (4.3)	10 426 (7.4)	29 319 (5.8)
Back pain	115 111 (17.7)	65 675 (31.3)	49 436 (11.3)	40 658 (28.7)	74 453 (14.7)
Neck pain	34 530 (5.3)	17 741 (8.4)	16 789 (3.8)	12 356 (8.7)	22 174 (4.4)
Arthritis	326 377 (50.3)	147 814 (70.3)	178 563 (40.7)	99 055 (70.0)	227 322 (44.8)
Headache	3575 (0.6)	2157 (1.0)	1418 (0.3)	1300 (0.9)	2275 (0.4)
HIV	627 (0.1)	264 (0.1)	363 (0.1)	193 (0.1)	434 (0.1)

Abbreviations: CKD, chronic kidney disease; NSAIDs, nonsteroidal anti-inflammatory drugs.

^a^ Significant group differences were observed for all of the characteristics, assessed by *t* test and χ^2^ test (*P* < .001).

**Figure 1.**
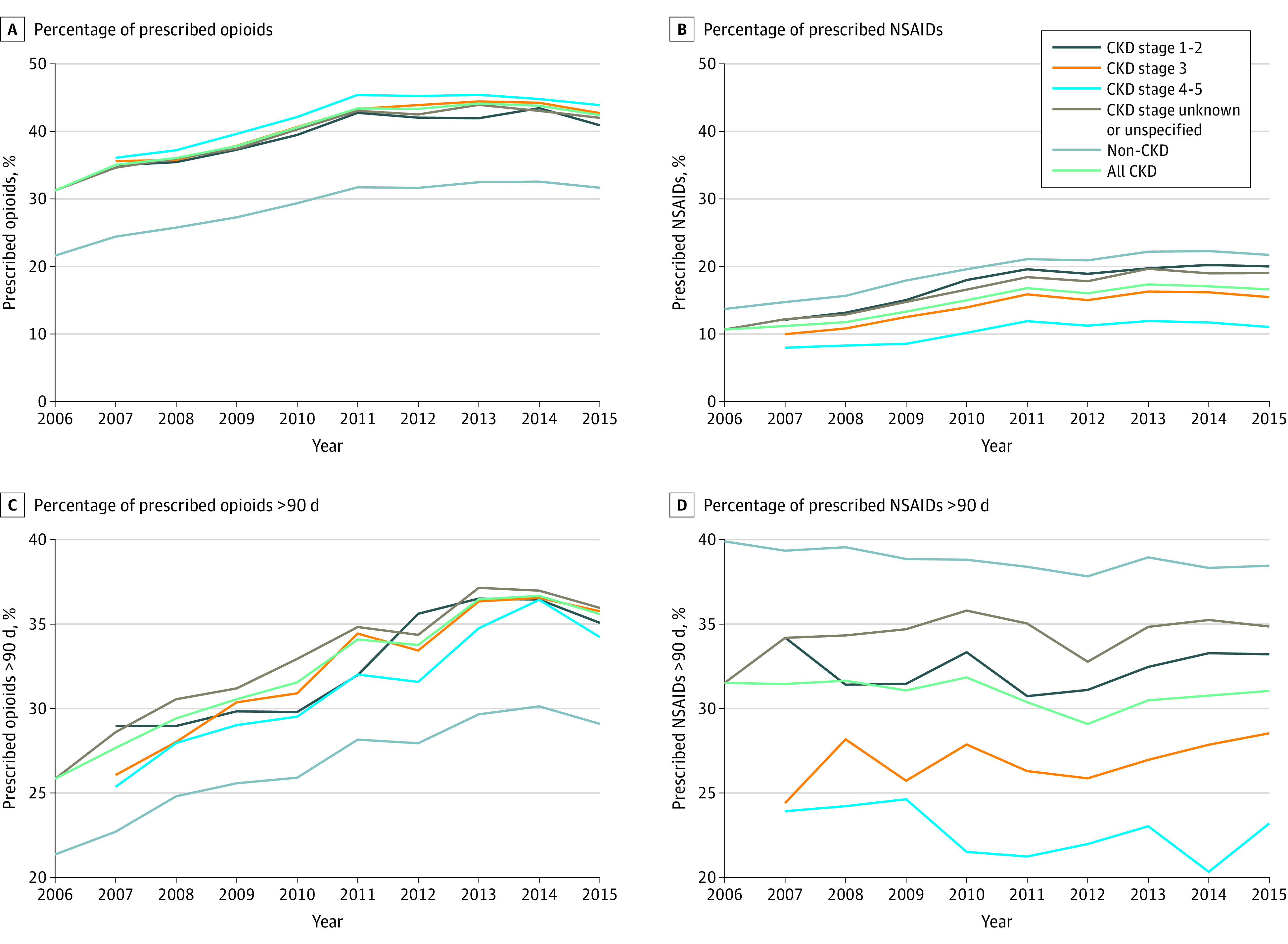
Trends in Use of Opioids and Prescription Nonsteroidal Anti-Inflammatory Drugs (NSAIDs) by Chronic Kidney Disease (CKD) Status, Annual Cohort 2006-2015 A, Opioids were prescribed to 31.2% of older patients with CKD in 2006, peaking at 44.1% in 2013 and then decreasing each year through 2015 (42.4%). B, Overall use of prescription NSAIDs increased from 10.7% in 2006 to a peak of 17.4% in 2013, with a gradual decrease to 16.6% in 2015. C, The proportion of prescribed opioids for more than 90 days was higher in the CKD users compared with the non-CKD users (35.6% vs 29.1%, 2015). D, Approximately one-third of CKD users had received prescription NSAIDs for more than 90 days, varying from 23.2% in CKD stages 4-5 to 33.2% in CKD stages 1-2 (2015).

Opioids were prescribed to 31.2% of older CKD patients in 2006, peaking at 44.1% in 2013 and then decreasing each year through 2015 (42.4%) (Figure 1A). Overall use of prescription NSAIDs increased from 10.7% in 2006 to a peak of 17.4% in 2013, with a gradual decrease to 16.6% in 2015 (Figure 1B). Opioid use was lowest in the non-CKD population (31.6% in 2015) and highest in CKD stages 4-5 (43.9% in 2015). Unlike with opioids, a lower percentage of patients with CKD received prescription NSAIDs compared with the non-CKD population (16.6% vs 21.7% in 2015), and the proportion was consistently lower at higher (more advanced) stages of CKD. In time-trend analyses, there was a significant dose-response relationship between CKD stage and opioid use after adjustment for time and demographic characteristics (non-CKD is the reference group; HR of CKD stages 1-2, 1.46 [95% CI, 1.38-1.53]; HR of CKD stage 3, 1.55 [95% CI, 1.50-1.59]; and HR of CKD stages 4-5, 1.67 [95% CI, 1.60-1.75]), while an inverse association was found in NSAID use (eTable 4 in the Supplement). Long-term use of opioids and prescribed opioids for more than 90 days increased during 2006-2014 (25.8%-36.7%) but decreased through 2015 at 35.6% (Figure 1C). The proportion of prescribed opioids for more than 90 days was higher in the CKD users compared with the non-CKD users (35.6% vs 29.1%, 2015) (Figure 1C). Long-term use of NSAIDs was stable through 2015 (Figure 1D). Approximately one-third of CKD users had received prescription NSAIDs for more than 90 days, varying from 23.2% in stages 4-5 CKD to 33.2% in stages 1-2 CKD (2015) (Figure 1D).

In terms of cumulative use, despite a greater proportion of those with advanced CKD using prescription opioids (CKD stage 3 and stages 4-5: 42.7% and 43.9% in 2015), this group had lower cumulative opioid use (CKD stage 3 and CKD stages 4-5: 113.8 days and 108.4 days) (eFigure 3 in the Supplement). The non-CKD population and CKD patients at early stages had greater cumulative use of prescription NSAIDs (non-CKD and CKD stages 1-2: 123.9 and 103.7 days in 2015) than advanced cases of CKD (CKD stage 3 and stages 4-5: 94.5 and 79.1 days in 2015).

The proportions of prescribed opioids and NSAIDs varied by race and comorbidities. In Asian American patients, in contrast with White patients and Black patients, the lowest utilization rate for opioids (26.4% in 2015) but the highest utilization rate for prescription NSAIDs (28.7% in 2015) regardless of CKD status were noted (eFigure 4 in the Supplement). The proportion with opioid use was significantly higher among patients with comorbidities than among those without, regardless of comorbidity type and CKD status (eg, hypertension vs nonhypertension: 42.9% vs 36.8%; cardiovascular disease vs non–cardiovascular disease: 44.2% vs 37.9%; diabetes vs nondiabetes: 44.3% vs 40.5%; cancer vs noncancer: 44.6% vs 41.9%; HIV vs non-HIV: 51.1% vs 42.4%; headache vs nonheadache: 61.1% vs 42.2%; arthritis vs nonarthritis:47.7% vs 30.0%; neck pain vs non–neck pain: 57.5% vs 41.1%; back pain vs non–back pain: 59.1% vs 36.2%; and depression vs nondepression: 52.9% vs 40.4% in 2015 (eFigure 5 in the Supplement). In particular, headache, back pain, and neck pain showed higher usage rates compared with other comorbidities.

**Figure 2.**
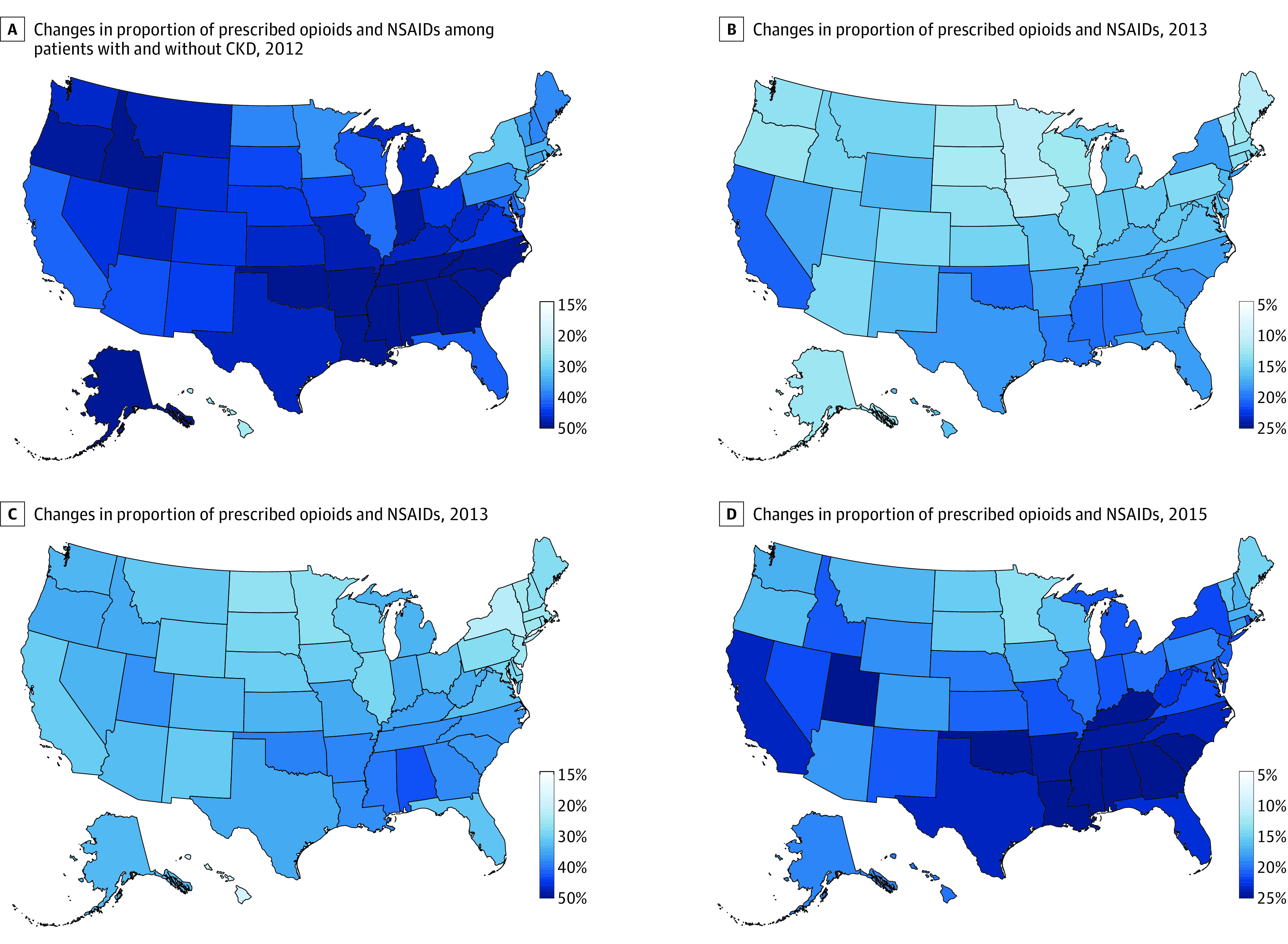
Changes in Proportion of Prescribed Opioids and Nonsteroidal Anti-Inflammatory Drugs (NSAIDs) Among Patients With and Without Chronic Kidney Disease (CKD), 2012-2015

Overall use of opioids and prescription NSAIDs varied widely across states from 2006 to 2015 (eg, range of opioid use in CKD: 21.8%-47.0% in 2006-2008, 28.0%-51.0% in 2009-2011, 24.7%-54.3% in 2012-2015; range of NSAID use in CKD: 7.4%-16.5% in 2006-2008, 9.3%-20.7% in 2009-2011; 11.2%-20.8% in 2012-2015) (eFigures 6 and 7 in the Supplement). As shown in Figure 2, the Rocky Mountain region and the Appalachian states had higher proportions of prescribed opioids from 2012 to 2015, while California, Utah and Southern states showed relatively higher proportions of prescribed NSAIDs. Geographic variation in long-term and cumulative use of opioids and prescription NSAIDs is shown in eFigures 8-11 in the Supplement. In comparing geographic variation of the 3 analgesic use measurements, states with high proportions of overall use were not necessarily the states with more long-term users. For example, Mississippi had relatively high overall use of opioids but fewer long-term users (2012-2015: 51.5% vs 33.7%). In contrast, Vermont had relatively low overall use of opioids but more long-term users (2012-2015: 36.8% vs 42.7%).

As shown in Table 2, opioid use was higher in patients with CKD, particularly stages 4-5 (odds ratio [OR], 1.35; 95% CI, 1.33-1.37; *P* < .001) compared with non-CKD with adjustment of demographic characteristics and CKD- and pain-related comorbidities. NSAID use was lower in patients with CKD stages 4-5 (OR, 0.55; 95% CI, 0.54-0.56; *P* < .001). There were some overlapping factors associated with higher odds of analgesic prescriptions (opioid or NSAID), like younger age (aged 65-75 years), female sex, hypertension, diabetes, back pain, neck pain, arthritis, headache, and HIV. In contrast, individuals who were White and had CKD, cancer, and cardiovascular disease were more likely to use opioids but less likely to use NSAIDs. Having depression was significantly associated with opioid use (OR, 1.25; 95% CI, 1.24-1.26; *P* < .001) but not prescription NSAID use (OR, 1.00; 95% CI, 0.99-1.01; *P* = .45) (Table 2). Factors associated with use of prescribed opioids and NSAIDs for more than 90 days are shown in eTable 5 in the Supplement.

**Table 2.** Results of Generalized Linear Models for the Association of Patients’ Baseline Characteristics With Opioid and Prescription NSAID Use in the Annual Cohort 2006-2015

Characteristic	Ever used opioids		Ever used prescription NSAIDs	
	OR (95% CI)	*P* value	OR (95% CI)	*P* value
Age group, y				
65 to <75	1 [Reference]	NA	1 [Reference]	NA
≥75 to <85	0.97 (0.97-0.97)	<.001	0.90 (0.90-0.91)	<.001
≥85	0.94 (0.93-0.95)	<.001	0.66 (0.66-0.67)	<.001
Sex				
Male	1 [Reference]	NA	1 [Reference]	NA
Female	1.16 (1.15-1.16)	<.001	1.22 (1.21-1.23)	<.001
Race/ethnicity				
White	1 [Reference]	NA	1 [Reference]	NA
Asian American	0.59 (0.58-0.60)	<.001	1.74 (1.71-1.78)	<.001
Other	0.79 (0.78-0.81)	<.001	1.18 (1.15-1.21)	<.001
Black	1.04 (1.03-1.05)	<.001	1.22 (1.20-1.23)	<.001
Unknown	0.86 (0.83-0.90)	<.001	1.22 (1.17-1.28)	<.001
Hypertension	1.16 (1.16-1.17)	<.001	1.17 (1.16-1.17)	<.001
Cardiovascular disease	1.17 (1.16-1.17)	<.001	0.88 (0.88-0.89)	<.001
Diabetes	1.20 (1.20-1.21)	<.001	1.09 (1.08-1.10)	<.001
CKD status				
Non-CKD	1 [Reference]	NA	1 [Reference]	NA
Stages 1-2	1.21 (1.19-1.24)	<.001	0.84 (0.82-0.86)	<.001
Stage 3	1.30 (1.29-1.31)	<.001	0.71 (0.70-0.72)	<.001
Stages 4-5	1.35 (1.33-1.37)	<.001	0.55 (0.54-0.56)	<.001
Unknown/other	1.12 (1.11-1.13)	<.001	0.80 (0.80-0.81)	<.001
Cancer	1.16 (1.15-1.17)	<.001	0.94 (0.93-0.95)	<.001
Depression	1.25 (1.24-1.26)	<.001	1.00 (0.99-1.01)	.45
Back pain	1.46 (1.45-1.46)	<.001	1.24 (1.23-1.24)	<.001
Neck pain	1.09 (1.08-1.10)	<.001	1.12 (1.11-1.13)	<.001
Arthritis	1.37 (1.37-1.38)	<.001	1.38 (1.37-1.38)	<.001
Headache	1.36 (1.33-1.39)	<.001	1.26 (1.22-1.29)	<.001
HIV	1.35 (1.28-1.42)	<.001	1.19 (1.12-1.26)	<.001

Abbreviations: CKD, chronic kidney disease; NA, not applicable; NSAIDs, nonsteroidal anti-inflammatory drugs; OR, odds ratio.

### Analgesic Use and Patient Outcomes

Approximately 32.4% and 21.8% of the longitudinal cohort had received opioids and prescription NSAIDs at baseline (Table 2). Opioid use, regardless of cumulative medication duration, was significantly associated with developing ESKD (HR, 1.10; 95% CI, 1.04-1.16; *P* < .001; accounting for the competing risk of death), independent of demographic characteristics, CKD status, and other investigated comorbidities (Table 3 and eTable 6 in the Supplement). There was no significant association between ever used prescription NSAIDs and developing ESKD, but those who received NSAIDs for 91 to 180 days were less likely to develop ESKD compared with non–NSAID users (HR, 0.77; 95% CI, 0.65-0.91; *P* < .001) (eTable 6 in the Supplement). Individuals who received opioids, regardless of short-term or long-term use, had a higher risk of death compared with non–opioid users, after adjusting for demographic characteristics and comorbidities (HR, 1.19; 95% CI, 1.18-1.20; *P* < .001). In contrast to opioid use, individuals who received prescription NSAIDs were at lower risk of death compared with non–NSAID users (HR, 0.84; 95% CI, 0.83-0.85; *P* < .001).

**Table 3.** Multivariable-Adjusted Cox Proportional Hazards Regression Models for Opioid and Prescription NSAID Use and CKD Outcomes

Characteristic	ESKD^a^		All-cause mortality	
	HR (95% CI)	*P* value	HR (95% CI)	*P* value
Ever used opioids	1.10 (1.04-1.16)	.001	1.19 (1.18-1.20)	<.001
Ever used prescription NSAIDs	0.95 (0.89-1.01)	.12	0.84 (0.83-0.85)	<.001
Age group, y				
65 to <75	1 [Reference]	NA	1 [Reference]	NA
≥75 to <85	0.82 (0.78-0.87)	<.001	2.13 (2.11-2.16)	<.001
≥85	0.43 (0.39-0.49)	<.001	5.22 (5.16-5.28)	<.001
Sex				
Male	1 [Reference]	NA	1 [Reference]	NA
Female	0.75 (0.71-0.79)	<.001	0.80 (0.79-0.81)	<.001
Race/ethnicity				
White	1 [Reference]	NA	1 [Reference]	NA
Asian American	1.68 (1.50-1.89)	<.001	0.65 (0.63-0.66)	<.001
Other	1.48 (1.27-1.73)	<.001	0.84 (0.81-0.86)	<.001
Black	2.08 (1.95-2.22)	<.001	1.06 (1.05-1.07)	<.001
Unknown	0.83 (0.45-1.55)	.56	0.92 (0.85-1.00)	.046
Hypertension	1.50 (1.38-1.62)	<.001	0.97 (0.96-0.97)	<.001
Cardiovascular disease	1.32 (1.24-1.40)	<.001	1.63 (1.62-1.65)	<.001
Diabetes	2.24 (2.11-2.37)	<.001	1.25 (1.24-1.26)	<.001
CKD status				
Non-CKD	1 [Reference]	NA	1 [Reference]	NA
Stages 1-2	6.59 (5.44-7.98)	<.001	1.36 (1.30-1.43)	<.001
Stage 3	10.81 (9.88-11.83)	<.001	1.41 (1.37-1.44)	<.001
Stages 4-5	51.17 (47.62-54.99)	<.001	2.04 (1.98-2.10)	<.001
Unknown/other	5.69 (5.23-6.19)	<.001	1.56 (1.53-1.58)	<.001
Cancer	1.01 (0.93-1.10)	.81	1.29 (1.28-1.31)	<.001
Depression	0.81 (0.72-0.92)	<.001	1.67 (1.65-1.70)	<.001
Back pain	0.88 (0.81-0.95)	.001	0.86 (0.85-0.86)	<.001
Neck pain	0.92 (0.80-1.05)	.20	0.81 (0.80-0.83)	<.001
Arthritis	0.82 (0.78-0.87)	<.001	1.11 (1.11-1.12)	<.001
Headache	0.72 (0.47-1.11)	.14	0.73 (0.69-0.78)	<.001
HIV	1.34 (0.74-2.42)	.34	1.65 (1.46-1.86)	<.001

Abbreviations: CKD, chronic kidney disease; ESKD, end-stage kidney disease; HR, hazard ratio; NA, not applicable; NSAID, nonsteroidal anti-inflammatory drug.

^a^ A competing risk model (accounting for death) was used to estimate the HR for development of ESKD.

## Discussion

To our knowledge, this is the largest investigation of real-world use of prescription analgesics among older adults with and without CKD in the US, covering 2006 to 2015. Use of analgesics, particularly opioids, was high in the Medicare CKD population, increasing substantially between 2006 and 2013, then stabilizing or decreasing slightly thereafter. A higher proportion of opioid vs prescription NSAID use was found in patients with CKD compared with non-CKD Medicare beneficiaries. As expected, cumulative use of analgesics, both opioids and prescription NSAIDs, decreased with CKD progression. This prescription pattern could be associated with patients with advanced CKD being more susceptible to toxic effects and adverse effects in the setting of reduced kidney function. Additionally, NSAIDs were widely used in earlier stages of CKD while opioids were more frequently used in patients with advanced CKD. This finding may reflect both the variation in severity of pain by CKD stage, as opioids are typically used to treat moderate to severe pain, and avoidance of NSAIDs in patients with more advanced CKD, in whom risk of progression is higher with use of this class of medication owing to the inhibitory effect of NSAIDs on prostaglandin synthesis in the kidneys, with resultant potential for intrakidney ischemia.^2^

The trends in opioid use observed in this study are consistent with a recent study of the general US population by the Centers for Disease Control and Prevention using retail prescription data.^19^ The fluctuation in analgesic use from 2011 to 2013 and the decrease after 2013 in our study may be associated with the effectiveness of federal regulations, state activities, local interventions, and heightened public awareness of the risks of opioid use.^20^ Dowell et al^21^ found that the usage rate of opioids and opioid overdose death rates decreased by 8 and 12 percentage points after implementation of statewide Prescription Drug Monitoring Program and pain clinic laws during 2011 and 2013. Similarly, 2 studies in Florida revealed that drug overdose deaths decreased substantially throughout the period from 2010 to 2012 owing to opioid-related regulations.^22,23^

The racial/ethnic differences in analgesic use observed in our study may be associated with barriers in accessing health care and different cultural perceptions of pain management. For example, Asian American patients used more prescription NSAIDs than opioids compared with other racial/ethnic groups. A prior study found that Asian American patients had greater perceived barriers to cancer pain management than patients of other races/ethnicities, which were consistent with their concerns about disease progression, drug tolerance, and addiction and view of cancer pain as inevitable.^24^ With respect to comorbidities, our findings were consistent with previous findings that the presence of certain comorbidities such as diabetes, hypertension, depression, cancer, back pain, neck pain, arthritis, and headache increased patients’ need for pain relief medications.^25,26,27^ High rates of comorbid depression indicate a substantial burden of mental health problems in the CKD population.^17^ Patients with comorbid CVD were less likely to use NSAIDs, which may be due to the cardiovascular risks associated with these medications.^28,29^

The substantial geographic variations in overall, long-term, and cumulative use of opioids and NSAIDs may reflect inconsistent treatment strategies across states. States with the highest prevalence of opioid or NSAID use did not necessarily have the longest average cumulative use of these medications. Previous studies also showed geographic variations in opioid use and opioid-related hospitalization and emergency department visits.^19,30^ These geographic variations suggest that each state or county may be facing different issues regarding inappropriate use of prescription analgesics. Considering the epidemic of opioid misuse, overdose, and death, it would be particularly relevant to design local regulations, educate physicians regarding opioid therapies, and raise public awareness about the risks of opioid use.^31^

In our outcomes analyses, opioid use was associated with a higher risk of developing ESKD and death, independent of CKD status, which may be due to opioid users being at high risk of opioid dependence and opioid overdose, with consequent higher hospitalization and mortality.^9,31^ It is possible that people in pain requested or received more opioids. In contrast with opioids, no association was observed between NSAID use (except receiving NSAIDs for 91-180 days) and progression to ESKD, but a significant protective association was observed between receiving NSAIDs and death, with adjustment of other patient-level factors. Previous studies have been inconsistent with regard to the association between NSAIDs and progression to ESKD. Some found that regular consumption of NSAIDs was associated with decreased kidney function.^32,33,34,35^ Additionally, the risk of developing ESKD increased with greater cumulative exposure to NSAIDs among those with CKD.^36,37^ However, others have suggested that use of NSAIDs was not associated with decreased kidney function.^38,39,40,41,42^ Use of NSAIDs in CKD requires caution because of their known direct nephrotoxic effects, risk of causing fluid and electrolyte imbalances, hypertension, and other complications.^2,6^ However, effective pain management may relieve pain and related stress and could potentially delay disease progression and mortality.^1^ The observed protective association of NSAIDs and outcomes may reflect the benefits of pain relief relative to potential nephrotoxic effects, or it may be the consequence of rational clinical decision-making, such that physicians use NSAIDs for patients with CKD at earlier stages and for those who have low risk of progressing to ESKD regardless of stage, while they choose opioids for patients at later stages of disease and high risk of progression. Our results highlight the need for further investigation of the benefits and risks of choosing NSAIDs vs opioids in pain management.

### Limitations

This study has limitations. First, diagnosis of CKD and other comorbidities was determined using health care claims data, with potential for underrecognition and undercoding of these conditions. Owing to lack of laboratory data, we used stage-specific *ICD-9-CM* codes (implemented in October 2005) to assess CKD stage. Thus, the accurate and complete coding for CKD in general and by stage likely improved over our study period, which could affect the observed trends.^43^ Further analysis of use of opioids and prescription NSAIDs in CKD, particularly in patients with stage 1 and stage 2 CKD identified by presence of albuminuria and proteinuria, would have been more informative; however, we do not have laboratory data available in Medicare claims data sets. Second, analgesic use was measured from medication claims and may not reflect actual use of medication. Medication claims cannot capture prescriptions filled outside the Medicare Part D networks. Most NSAID use is over the counter, and NSAID use reported in our study is likely a fraction of actual NSAID use. Third, substantial limits on opioid use in response to the opioid crisis have been implemented since 2015, so our trends may not reflect the most recent changes in opioid use. Fourth, our study may be subject to omitted variable bias, as not all of the risk factors that may be associated with pain and CKD progression were controlled for. Last, we restricted our study patients to fee-for-service Medicare beneficiaries enrolled in Part D owing to lack of data from Medicare Advantage plans. Thus, our results may not be generalizable to other populations. Additionally, Medicare Part D enrollment increased over time, which may also be a factor in the observed increase in analgesic use.

## Conclusions

Our study suggests that there has been substantial use of opioids and prescription NSAIDs among older patients with CKD in the US with a clear increasing trend until recently, with early suggestion of decreasing use, perhaps due to the recent heightened awareness of the opioid epidemic in the US. Clinicians must continue evaluating benefits and risks of using opioids and NSAIDs in this and other patient populations. Specifically, clear clinical guidelines for chronic pain management among patients aged 65 years and older with CKD may be warranted to address potentially suboptimal pain management in this patient population.

## Notes

Supplement. **eFigure 1.** Study Design and Sample Selection for the Yearly Cohort 2006-2015 **eFigure 2.** Study Design and Sample Selection for the Longitudinal Cohort **eTable 1.** ICD-9-CM Codes Used to Define CKD and Other Comorbidities **eTable 2.** Baseline Characteristics of the Yearly Cohort by Year (2006-2015) **eTable 3.** Baseline Characteristics of the Yearly Cohort by Prescription Analgesics Use Status and CKD Status (2006-2015) **eFigure 3.** Trends in Total Annual Days' Supply of Opioids and Prescription NSAIDs by CKD Status, the Yearly Cohort 2006-2015 **eFigure 4.** Trends in Proportion of Prescribed Opioids and NSAIDs by CKD and Race, the Yearly Cohort 2006-2015 **eFigure 5.** Trends in Use of Opioids and Prescription NSAIDs by CKD and Selected Comorbidities, the Yearly Cohort 2006-2015 **eTable 4.** Results of Trend Analyses for Opioid and Prescription NSAID Use According to CKD Status, the Yearly Cohort 2006-2015 **eFigure 6.** Changes in Proportion of Prescribed Opioids Among CKD and Without CKD From Year 2006-2015 **eFigure 7.** Changes in Proportion of Prescribed NSAIDs Among CKD and Without CKD From Year 2006-2015 **eFigure 8.** Changes in Proportion of Prescribed Opioids Greater Than 90 Days Among Users With CKD and Without CKD From 2006-2015 **eFigure 9.** Changes in Proportion of Prescribed NSAIDs Greater Than 90 Days Among Users With CKD and Without CKD From 2006-2015 **eFigure 10.** Changes in Total Annual Days' Supply of Opioids Among Users With CKD and Without CKD From 2006-2015 **eFigure 11.** Changes in Total Annual Days' Supply of Prescription NSAIDs Among Users With CKD and Without CKD From 2006-2015 **eTable 5.** Results of Generalized Linear Models for the Relationship of Patients’ Baseline Characteristics to Opioid and Prescription NSAID Use (as Prescribed Greater Than 90 Days), the Yearly Cohort 2006-2015 **eTable 6.** Results of Multivariable-Adjusted Cox Models for the Association of Opioid and Prescription NSAID Use (as Total Annual Days' Supply) With CKD Outcomes
