# Supplementary material for: Assessment of Prescription Analgesic Use in Older Adults With and Without Chronic Kidney Disease and Outcomes
Source: JAMA Netw Open. 2020 Sep 30;3(9):e2016839. doi: 10.1001/jamanetworkopen.2020.16839 (PMC7527874; doi:10.1001/jamanetworkopen.2020.16839)
Supplement: Supplement. — eFigure 1. Study Design and Sample Selection for the Yearly Cohort 2006-2015 eFigure 2. Study Design and Sample Selection for the Longitudinal Cohort eTable 1. ICD-9-CM Codes Used to Define CKD and Other Comorbidities eTable 2. Baseline Characteristics of the Yearly Cohort by Year (2006-2015) eTable 3. Baseline Characteristics of the Yearly Cohort by Prescription Analgesics Use Status and CKD Status (2006-2015) eFigure 3. Trends in Total Annual Days' Supply of Opioids and Prescription NSAIDs by CKD Status, the Yearly Cohort 2006-2015 eFigure 4. Trends in Proportion of Prescribed Opioids and NSAIDs by CKD and Race, the Yearly Cohort 2006-2015 eFigure 5. Trends in Use of Opioids and Prescription NSAIDs by CKD and Selected Comorbidities, the Yearly Cohort 2006-2015 eTable 4. Results of Trend Analyses for Opioid and Prescription NSAID Use According to CKD Status, the Yearly Cohort 2006-2015 eFigure 6. Changes in Proportion of Prescribed Opioids Among CKD and Without CKD From Year 2006-2015 eFigure 7. Changes in Proportion of Prescribed NSAIDs Among CKD and Without CKD From Year 2006-2015 eFigure 8. Changes in Proportion of Prescribed Opioids Greater Than 90 Days Among Users With CKD and Without CKD From 2006-2015 eFigure 9. Changes in Proportion of Prescribed NSAIDs Greater Than 90 Days Among Users With CKD and Without CKD From 2006-2015 eFigure 10. Changes in Total Annual Days' Supply of Opioids Among Users With CKD and Without CKD From 2006-2015 eFigure 11. Changes in Total Annual Days' Supply of Prescription NSAIDs Among Users With CKD and Without CKD From 2006-2015 eTable 5. Results of Generalized Linear Models for the Relationship of Patients’ Baseline Characteristics to Opioid and Prescription NSAID Use (as Prescribed Greater Than 90 Days), the Yearly Cohort 2006-2015 eTable 6. Results of Multivariable-Adjusted Cox Models for the Association of Opioid and Prescription NSAID Use (as Total Annual Days' Supply) With CKD Outcomes [file jamanetwopen-e2016839-s001.pdf]

Supplemental Online Content

Han Y, Balkrishnan R, Hirth RA, et al. Assessment of prescription analgesic use in older adults with and without chronic kidney disease and outcomes. *JAMA Netw Open*. 2020;3(9):e2016839. doi:10.1001/jamanetworkopen.2020.16839

eFigure 1. Study Design and Sample Selection for the Yearly Cohort 2006-2015

eFigure 2. Study Design and Sample Selection for the Longitudinal Cohort

eTable 1. ICD-9-CM Codes Used to Define CKD and Other Comorbidities

eTable 2. Baseline Characteristics of the Yearly Cohort by Year (2006-2015)

eTable 3. Baseline Characteristics of the Yearly Cohort by Prescription Analgesics Use Status and CKD Status (2006-2015)

eFigure 3. Trends in Total Annual Days' Supply of Opioids and Prescription NSAIDs by CKD Status, the Yearly Cohort 2006-2015

eFigure 4. Trends in Proportion of Prescribed Opioids and NSAIDs by CKD and Race, the Yearly Cohort 2006-2015

eFigure 5. Trends in Use of Opioids and Prescription NSAIDs by CKD and Selected Comorbidities, the Yearly Cohort 2006-2015

eTable 4. Results of Trend Analyses for Opioid and Prescription NSAID Use According to CKD Status, the Yearly Cohort 2006-2015

eFigure 6. Changes in Proportion of Prescribed Opioids Among CKD and Without CKD From Year 2006-2015

eFigure 7. Changes in Proportion of Prescribed NSAIDs Among CKD and Without CKD From Year 2006-2015

eFigure 8. Changes in Proportion of Prescribed Opioids Greater Than 90 Days Among Users With CKD and Without CKD From 2006-2015

eFigure 9. Changes in Proportion of Prescribed NSAIDs Greater Than 90 Days Among Users With CKD and Without CKD From 2006-2015

eFigure 10. Changes in Total Annual Days' Supply of Opioids Among Users With CKD and Without CKD From 2006-2015

© 2020 Han Y et al. *JAMA Network Open*.

eFigure 11. Changes in Total Annual Days' Supply of Prescription NSAIDs Among Users With CKD and Without CKD From 2006-2015

eTable 5. Results of Generalized Linear Models for the Relationship of Patients' Baseline Characteristics to Opioid and Prescription NSAID Use (as Prescribed Greater Than 90 Days), the Yearly Cohort 2006-2015

eTable 6. Results of Multivariable-Adjusted Cox Models for the Association of Opioid and Prescription NSAID Use (as Total Annual Days' Supply) With CKD Outcomes

This supplemental material has been provided by the authors to give readers additional information about their work.

© 2020 Han Y et al. *JAMA Network Open*.

eFigure 1. Study design and sample selection for the yearly cohort 2006-2015

Study design

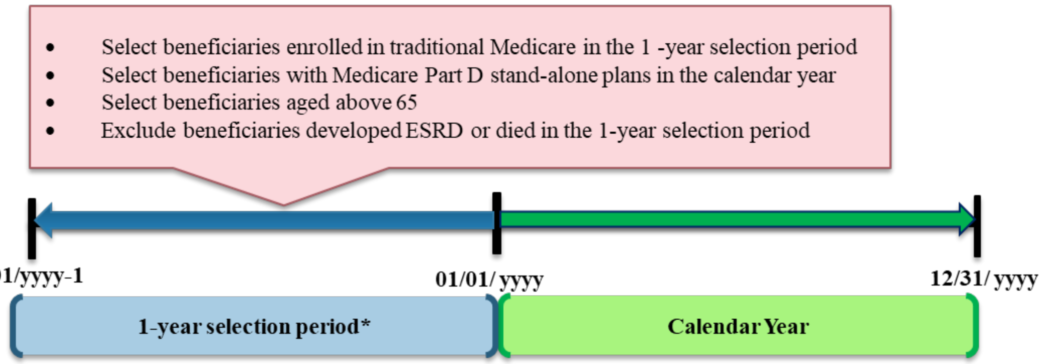

1-year selection period—assess demographics, CKD stages and comorbidities
Calendar year—assess analgesic use

Diagram of sample selection

	2006	2007	2008	2009	2010	2011	2012	2013	2014	2015
All Medicare beneficiaries without ESRD	1,497,282	1,448,250	1,425,400	1,413,026	1,412,821	1,430,802	1,439,659	1,441,563	1,480,857	1,470,831
↓ Continuously enrolled in Part D in the 1-year selection period	752,610	749,280	757,816	758,574	769,683	797,519	730,077	946,390	982,336	986,898
↓ Exclude Medicare beneficiaries covered by Medicare Advantage plans and aged below 65	553,862	562,952	569,260	573,495	582,654	607,080	536,601	738,294	759,012	777,244

© 2020 Han Y et al. *JAMA Network Open*.

eFigure 2. Study design and sample selection for the longitudinal cohort

Study design

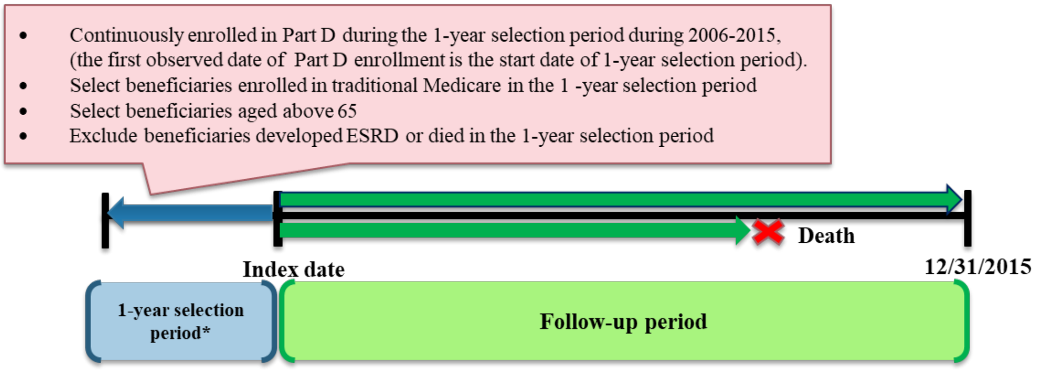

1-year selection period—assess demographics, CKD stages, comorbidities and analgesic use

Diagram of sample selection

Select Medicare beneficiaries with Part D coverage in 2006-2015. The first observed date of Part D enrollment is the start date of 1-year selection period.
N=1,653,978

↓

Exclude Medicare beneficiaries covered by Medicare Advantage plans
N=1,093,611

↓

Exclude Medicare beneficiaries aged below 65
N=697,122

↓

Exclude Medicare beneficiaries who died or got ESRD in the 1-year selection period
N=649,339

© 2020 Han Y et al. *JAMA Network Open*.

eTable 1. ICD-9-CM codes used to define CKD and other comorbidities

	ICD-9-CM codes
Chronic kidney disease (CKD)	016.0; 095.4; 189.0,189.9; 223.0; 236.91; 250.4; 271.4; 274.1; 283.11; 403; 404; 440.1; 442.1; 477.3; 572.4; 581-583; 585- 588; 591; 642.1; 646.2; 753.12-753.19; 753.2; 794.4
Staging of chronic kidney disease	
Stage 1	585.1
Stage 2	585.2
Stage 3	585.3
Stage 4	585.4
Stage 5	585.5 or 585.6 with no CMS 2728 form
Stage unknown or unspecified	Patient has no claims with codes 585.1- 585.6 but has: 016.0; 095.4; 189.0,189.9; 223.0; 236.91; 250.4; 271.4; 274.1; 283.11; 403; 404; 440.1; 442.1; 477.3; 572.4; 581- 583; 585.9; 586-588; 591; 642.1; 646.2; 753.12-753.19; 753.2; 794.4
Hypertension (HTN)	362.11; 401-405; 437.2
Cardiovascular disease (CVD)	398.91; 402.01, 402.11, 402.91; 404.01, 404.03, 404.11, 404.13, 404.91, 404.93; 410-414; 422; 425-428; 430-438; 440-444; 447; 451-453; 557; V42.1, V45.0, V45.81, V45.82, V53.3
Diabetes mellitus (DM)	250; 357.2; 362.0; 366.41
Cancer	140-172; 174-208; 230-231; 233-234
Depression	296.2, 311
Back pain	721.3x - 721.9x, 722.2x, 722.30, 722.70, 722.80, 722.90, 722.32, 722.72, 722.82, 722.92, 722.33, 722.73, 722.83, 722.93, 724.xx, 737.1, 737.3, 738.4, 738.5, 739.2, 739.3, 739.4, 756.10, 756.11, 756.12, 756. 13, 756.19, 805.4, 805.8, 839.2, 839.42, 846, 846.0, 847.1, 847.3, 847.2, 847.9
Neck pain	721.0X, 721.1X, 722.0X, 722.31, 722.71, 722.81, 722.91, 723.XX, 839.0, 839.1, 847. 0
Arthritis/joint pain	710≤codes <720,725≤codes <740
Headache/migraine	346≤codes <347, 307.81
Human immunodeficiency virus (HIV)	042
Source: ICD-9-CM, International Classification of Diseases, Ninth/Tenth Revision, Clinical Modification. ICD-9-CM diagnosis codes can have up to five digits with a decimal point between the 3rd and 4th digits, while ICD-10-CM codes have seven digits. Codes listed with three digits include all existing 4th and 5th digits, and those listed with four digits include all existing 5th digits.	

© 2020 Han Y et al. *JAMA Network Open*.

eTable 2. Baseline characteristics of the yearly cohort by year (2006-2015)

Characteristics	2006 (n=553862)	2007 (n=562952)	2008 (n=569260)	2009 (n=573495)	2010 (n=582654)	2011 (n=607080)	2012 (n=536601)	2013 (n=738294)	2014 (n=759012)	2015 (n=777244)
Use of Opioids										
Ever use	431408 (77.9%)	421469 (74.9%)	418249 (73.5%)	411971 (71.8%)	405495 (69.6%)	407323 (67.1%)	360214 (67.1%)	488980 (66.2%)	502040 (66.1%)	521256 (67.1%)
Total annual days' supply (mean, sd)	122454 (22.1%)	141483 (25.1%)	151011 (26.5%)	161524 (28.2%)	177159 (30.4%)	199757 (32.9%)	176387 (32.9%)	249314 (33.8%)	256972 (33.9%)	255988 (32.9%)
Total annual days' supply categories	14.4 (54.4)	17.8 (62.9)	20.8 (70.5)	23.0 (75.4)	25.7 (80.1)	30.4 (89.6)	30.6 (91.6)	33.7 (97.1)	34.7 (99.9)	33.0 (97.6)
0 days	431416 (77.9%)	421469 (74.9%)	418250 (73.5%)	411971 (71.8%)	405495 (69.6%)	407323 (67.1%)	360214 (67.1%)	488980 (66.2%)	502040 (66.1%)	521256 (67.1%)
1-90 days	95873 (17.3%)	108689 (19.3%)	112831 (19.8%)	119288 (20.8%)	130005 (22.3%)	141899 (23.4%)	125644 (23.4%)	172872 (23.4%)	177002 (23.3%)	178926 (23.0%)
91-180 days	12209 (2.2%)	13926 (2.5%)	14987 (2.6%)	16277 (2.8%)	17773 (3.1%)	21008 (3.5%)	17913 (3.3%)	25747 (3.5%)	25845 (3.4%)	24522 (3.2%)
>180 days	14364 (2.6%)	18868 (3.4%)	23192 (4.1%)	25959 (4.5%)	29381 (5.0%)	36850 (6.1%)	32830 (6.1%)	50695 (6.9%)	54125 (7.1%)	52540 (6.8%)
Use of NSAIDs										
Ever use	478843 (86.5%)	481372 (85.5%)	481856 (84.6%)	472941 (82.5%)	471047 (80.8%)	481754 (79.4%)	427219 (79.6%)	578502 (78.4%)	594501 (78.3%)	613266 (78.9%)
Total annual days' supply (mean, sd)	75019 (13.5%)	81580 (14.5%)	87404 (15.4%)	100554 (17.5%)	111607 (19.2%)	125326 (20.6%)	109382 (20.4%)	159792 (21.6%)	164511 (21.7%)	163978 (21.1%)
Total annual days' supply categories	15.5 (56.1)	17.1 (61.3)	18.5 (64.2)	20.7 (67.6)	22.8 (71.3)	24.4 (74.1)	23.9 (73.1)	26.4 (78.1)	26.2 (77.8)	25.7 (77.2)
0 days	478861 (86.5%)	481373 (85.5%)	481856 (84.6%)	472943 (82.5%)	471047 (80.8%)	481754 (79.4%)	427219 (79.6%)	578502 (78.4%)	594501 (78.3%)	613266 (78.9%)
1-90 days	45338 (8.2%)	49821 (8.8%)	53240 (9.4%)	61992 (10.8%)	68867 (11.8%)	78052 (12.9%)	68814 (12.8%)	98776 (13.4%)	102605 (13.5%)	102074 (13.1%)
91-180 days	12644 (2.3%)	11834 (2.1%)	12438 (2.2%)	14181 (2.5%)	15517 (2.7%)	17057 (2.8%)	14626 (2.7%)	21320 (2.9%)	21601 (2.8%)	21435 (2.8%)
>180 days	17019 (3.1%)	19924 (3.5%)	21726 (3.8%)	24379 (4.3%)	27223 (4.7%)	30217 (5.0%)	25942 (4.8%)	39696 (5.4%)	40305 (5.3%)	40469 (5.2%)
Demographics										
Age (mean, sd)	77.0 (7.7)	77.1 (7.7)	77.1 (7.8)	77.0 (7.8)	77.0 (7.8)	76.9 (7.8)	76.2 (7.5)	76.6 (7.8)	76.5 (7.8)	76.3 (7.7)
Age group										
65-75	249860 (45.1%)	253740 (45.1%)	259829 (45.6%)	266329 (46.4%)	273995 (47.0%)	288781 (47.6%)	277622 (51.7%)	362293 (49.1%)	380186 (50.1%)	397497 (51.1%)
75-85	210653 (38.0%)	210865 (37.5%)	209221 (36.8%)	206290 (36.0%)	205963 (35.3%)	212086 (34.9%)	180203 (33.6%)	251728 (34.1%)	254286 (33.5%)	256060 (32.9%)
85 and above	93349 (16.9%)	98347 (17.5%)	100210 (17.6%)	100876 (17.6%)	102696 (17.6%)	106213 (17.5%)	78776 (14.7%)	124273 (16.8%)	124540 (16.4%)	123687 (15.9%)
Male	187892 (33.9%)	194271 (34.5%)	199913 (35.1%)	203910 (35.6%)	210089 (36.1%)	222959 (36.7%)	233077 (43.4%)	285926 (38.7%)	298723 (39.4%)	309943 (39.9%)
Race										

© 2020 Han Y et al. *JAMA Network Open*.

White	470902 (85.0%)	480689 (85.4%)	487514 (85.6%)	490351 (85.5%)	497320 (85.4%)	517917 (85.3%)	467925 (87.2%)	634311 (85.9%)	655452 (86.4%)	674930 (86.8%)
Asian	11868 (2.1%)	12957 (2.3%)	13695 (2.4%)	14111 (2.5%)	14780 (2.5%)	15389 (2.5%)	8119 (1.5%)	16266 (2.2%)	15325 (2.0%)	14025 (1.8%)
Other	8349 (1.5%)	9476 (1.7%)	10069 (1.8%)	10545 (1.8%)	11112 (1.9%)	11648 (1.9%)	9852 (1.8%)	13890 (1.9%)	14220 (1.9%)	14451 (1.9%)
Black	47770 (8.6%)	44944 (8.0%)	43412 (7.6%)	43709 (7.6%)	44220 (7.6%)	46234 (7.6%)	39714 (7.4%)	56181 (7.6%)	55695 (7.3%)	54525 (7.0%)
Unknown	753 (0.1%)	702 (0.1%)	669 (0.1%)	623 (0.1%)	618 (0.1%)	864 (0.1%)	943 (0.2%)	2674 (0.4%)	4669 (0.6%)	6987 (0.9%)
HTN	335390 (60.6%)	348990 (62.0%)	358653 (63.0%)	369007 (64.3%)	380243 (65.3%)	400054 (65.9%)	346912 (64.6%)	483846 (65.5%)	494856 (65.2%)	501194 (64.5%)
CVD	240900 (43.5%)	248357 (44.1%)	252262 (44.3%)	256237 (44.7%)	262026 (45.0%)	273128 (45.0%)	236666 (44.1%)	324362 (43.9%)	329293 (43.4%)	333147 (42.9%)
DM	128195 (23.1%)	134789 (23.9%)	140856 (24.7%)	146339 (25.5%)	152553 (26.2%)	162038 (26.7%)	141304 (26.3%)	197045 (26.7%)	200720 (26.4%)	202582 (26.1%)
CKD status*										
All CKD	29843 (5.4%)	38495 (6.8%)	43501 (7.6%)	49206 (8.6%)	55071 (9.5%)	62438 (10.3%)	57512 (10.7%)	83261 (11.3%)	88636 (11.7%)	93755 (12.1%)
non-CKD	524019 (94.6%)	524457 (93.2%)	525759 (92.4%)	524289 (91.4%)	527583 (90.5%)	544642 (89.7%)	479089 (89.3%)	655033 (88.7%)	670376 (88.3%)	683489 (87.9%)
CKD stage 1-2	NA	3160 (0.6%)	3557 (0.6%)	3913 (0.7%)	4535 (0.8%)	5365 (0.9%)	5287 (1.0%)	7951 (1.1%)	8689 (1.1%)	9630 (1.2%)
CKD stage 3	NA	7926 (1.4%)	11282 (2.0%)	14652 (2.6%)	18684 (3.2%)	23119 (3.8%)	23682 (4.4%)	35922 (4.9%)	40164 (5.3%)	44931 (5.8%)
CKD stage 4-5	NA	4929 (0.9%)	5771 (1.0%)	6264 (1.1%)	6894 (1.2%)	7475 (1.2%)	6396 (1.2%)	9247 (1.3%)	9324 (1.2%)	9523 (1.2%)
Unknown /other	29843 (5.4%)	22480 (4.0%)	22891 (4.0%)	24377 (4.3%)	24958 (4.3%)	26479 (4.4%)	22147 (4.1%)	30141 (4.1%)	30459 (4.0%)	29671 (3.8%)
Cancer	51639 (9.3%)	55110 (9.8%)	57573 (10.1%)	59502 (10.4%)	60906 (10.5%)	64203 (10.6%)	60637 (11.3%)	80016 (10.8%)	83102 (10.9%)	85821 (11.0%)
Depression	31785 (5.7%)	33684 (6.0%)	35045 (6.2%)	38763 (6.8%)	41032 (7.0%)	44232 (7.3%)	39958 (7.4%)	60647 (8.2%)	63166 (8.3%)	66004 (8.5%)
Back pain	96678 (17.5%)	100631 (17.9%)	104436 (18.3%)	108306 (18.9%)	113674 (19.5%)	121333 (20.0%)	109985 (20.5%)	155345 (21.0%)	161062 (21.2%)	168390 (21.7%)
Neck pain	28430 (5.1%)	29451 (5.2%)	30632 (5.4%)	32258 (5.6%)	34564 (5.9%)	37388 (6.2%)	34610 (6.4%)	48795 (6.6%)	51159 (6.7%)	54433 (7.0%)
Arthritis	280154 (50.6%)	289048 (51.3%)	297386 (52.2%)	306512 (53.4%)	317708 (54.5%)	334688 (55.1%)	289959 (54.0%)	405898 (55.0%)	415710 (54.8%)	425963 (54.8%)
Headache	2494 (0.5%)	2630 (0.5%)	2819 (0.5%)	3058 (0.5%)	3252 (0.6%)	3662 (0.6%)	3616 (0.7%)	5650 (0.8%)	6313 (0.8%)	6893 (0.9%)
HIV	240 (0.0%)	375 (0.1%)	469 (0.1%)	604 (0.1%)	760 (0.1%)	919 (0.2%)	936 (0.2%)	1306 (0.2%)	1478 (0.2%)	1571 (0.2%)

*CKD status was not reported in the 2006 cohort as the stage-specific ICD-9-CM CKD codes was introduced in October 2015.
Abbreviations: Chronic kidney disease (CKD), hypertension (HTN), cardiovascular disease (CVD), diabetes (DM) and human immunodeficiency virus (HIV)

© 2020 Han Y et al. *JAMA Network Open*.

eTable 3. Baseline characteristics of the yearly cohort by prescription analgesics use status and CKD status (2006-2015)

	CKD (n=601,718)					Non-CKD (n=5,658,736)				
		Opioids		Prescription NSAIDs			Opioids		Prescription NSAIDs	
**Characteristics**	**All**	**User (n=246671)**	**non-User (n=355047)**	**User (n=92283)**	**non-User (n=509435)**	**All**	**User (n=1645378)**	**non-User (n=4013358)**	**User (n=1086870)**	**non-User (n=4571866)**
Use of Opioids										
Ever used	246671 (40.99%)	246671(100.00%)	0(0.00%)	52413(56.80%)	194258(38.13%)	1645378(29.08%)	1645378(100.00%)	0(0.00%)	504118(46.38%)	1141260(24.96%)
Total annual days' supply (mean, sd)	42.88 (90.32)	104.60(141.10)	0.00(0.00)	67.44(128.00)	38.46(98.33)	25.41(72.48)	87.40(134.40)	0.00(0.00)	44.13(105.50)	20.97(75.52)
Total annual days' supply categories										
0 days	355047(59.01%)	0(0.00%)	355047(100.00%)	39870(43.20%)	315178(61.87%)	4013358(70.92%)	0(0.00%)	4013358(100.00%)	582753(53.62%)	3430613(75.04%)
1-90 days	163774(27.22%)	163774(66.39%)	0(0.00%)	32517(35.24%)	131257(25.77%)	1199255(21.19%)	1199255(72.89%)	0(0.00%)	354845(32.65%)	844410(18.47%)
91-180 days	30269(5.03%)	30269(12.27%)	0(0.00%)	6996(7.58%)	23273(4.57%)	159938(2.83%)	159938(9.72%)	0(0.00%)	54441(5.01%)	105497(2.31%)
>180 days	52627(8.75%)	52627(21.33%)	0(0.00%)	12900(13.98%)	39727(7.80%)	286177(5.06%)	286177(17.39%)	0(0.00%)	94831(8.73%)	191346(4.19%)
Use of NSAIDs										
Ever used	92283(15.34%)	52413(21.25%)	39870(11.23%)	92283(100.00%)	0(0.00%)	1086870(19.21%)	504118(30.64%)	582752(14.52%)	1086870(100.00%)	0(0.00%)
Total annual days' supply (mean, sd)	15.18(56.91)	21.54(67.23)	10.76(48.47)	98.95(114.10)	0.00(0.00)	23.29(71.87)	38.89(91.30)	16.90(62.17)	121.30(124.60)	0.00(0.00)
Total annual days' supply categories										
0 days	509437(84.66%)	194259(78.75%)	315178(88.77%)	0(0.00%)	509435(100.00%)	4571885(80.79%)	1141264(69.36%)	3430621(85.48%)	0(0.00%)	4571866(100.00%)
1-90 days	63876(10.62%)	35589(14.43%)	28287(7.97%)	63876(69.22%)	0(0.00%)	665703(11.76%)	295508(17.96%)	370195(9.22%)	665703(61.25%)	0(0.00%)
91-180 days	11455(1.90%)	6892(2.79%)	4563(1.29%)	11455(12.41%)	0(0.00%)	151198(2.67%)	74544(4.53%)	76654(1.91%)	151198(13.91%)	0(0.00%)
>180 days	16950(2.82%)	9931(4.03%)	7019(1.98%)	16950(18.37%)	0(0.00%)	269950(4.77%)	134062(8.15%)	135888(3.39%)	269950(24.84%)	0(0.00%)
Demographics										
Age (mean, sd)	79.22(7.96)	78.43(7.88)	79.77(8.01)	77.79(7.59)	79.48(8.03)	76.48(7.68)	76.33(7.66)	76.55(7.69)	75.44(7.09)	76.73(7.79)
Age group										
65-75	206563(34.33%)	93846(38.05%)	112717(31.75%)	37728(40.88%)	168835(33.14%)	2803569(49.54%)	828183(50.33%)	1975386(49.22%)	593006(54.56%)	2210563(48.35%)
75-85	240962(40.05%)	97809(39.65%)	143153(40.32%)	36878(39.96%)	204084(40.06%)	1956393(34.57%)	562799(34.20%)	1393594(34.72%)	369188(33.97%)	1587205(34.72%)
85 and above	154193(25.63%)	55016(22.30%)	99177(27.93%)	17677(19.16%)	136516(26.80%)	898774(15.88%)	254396(15.46%)	644378(16.06%)	124676(11.47%)	774098(16.93%)
Male	260332(43.26%)	96512(39.13%)	163820(46.14%)	36097(39.12%)	224235(44.02%)	2086371(36.87%)	551777(33.53%)	1534594(38.24%)	350630(32.26%)	1735741(37.97%)
Race										
White	491202(81.63%)	203193(82.37%)	288009(81.12%)	71282(77.24%)	419920(82.43%)	4886109(86.35%)	1434850(87.20%)	3451259(85.99%)	900972(82.90%)	3985137(87.17%)
Asian	13650(2.27%)	3872(1.57%)	9778(2.75%)	3478(3.77%)	10172(2.00%)	122885(2.17%)	24068(1.46%)	98817(2.46%)	34579(3.18%)	88306(1.93%)
Other	10549(1.75%)	3740(1.52%)	6809(1.92%)	1940(2.10%)	8609(1.69%)	103063(1.82%)	24862(1.51%)	78201(1.95%)	21595(1.99%)	81468(1.78%)
Black	70579(11.73%)	29661(12.02%)	40918(11.52%)	11827(12.82%)	58752(11.53%)	405825(7.17%)	123510(7.51%)	282315(7.03%)	90413(8.32%)	315412(6.90%)
Unknown	1409(0.23%)	537(0.22%)	872(0.25%)	273(0.30%)	1136(0.22%)	18093(0.32%)	4488(0.27%)	13605(0.34%)	3742(0.34%)	14351(0.31%)
HTN	554992(92.23%)	230093(93.28%)	324899(91.51%)	85831(93.01%)	469161(92.09%)	3464153(61.22%)	1131810(68.79%)	2332343(58.11%)	732748(67.42%)	2731405(59.74%)

© 2020 Han Y et al. *JAMA Network Open*.

CVD	445316(74.01%)	189484(76.82%)	255832(72.06%)	65379(70.85%)	379937(74.58%)	2311062(40.84%)	793029(48.20%)	1518033(37.82%)	431366(39.69%)	1879696(41.11%)
DM	295996(49.19%)	126868(51.43%)	169128(47.64%)	47153(51.10%)	248843(48.85%)	1310425(23.16%)	448284(27.25%)	862141(21.48%)	284467(26.17%)	1025958(22.44%)
CKD status										
All CKD	601718(100.00%)	246671(100.00%)	355047(100.00%)	92283(100.00%)	509435(100.00%)	0(0.00%)	0(0.00%)	0(0.00%)	0(0.00%)	0(0.00%)
non-CKD	0(0.00%)	0(0.00%)	0(0.00%)	0(0.00%)	0(0.00%)	5658736(100.00%)	1645378 (100.00%)	4013358 (100.00%)	1086870 (100.00%)	4571866 (100.00%)
CKD stage 1-2	52087(8.66%)	21167(8.58%)	30920(8.71%)	9559(10.36%)	42528(8.35%)	0(0.00%)	0(0.00%)	0(0.00%)	0(0.00%)	0(0.00%)
CKD stage 3	220362(36.62%)	93273(37.81%)	127089(35.79%)	32977(35.73%)	187385(36.78%)	0(0.00%)	0(0.00%)	0(0.00%)	0(0.00%)	0(0.00%)
CKD stage 4-5	65823(10.94%)	28140(11.41%)	37683(10.61%)	6966(7.55%)	58857(11.55%)	0(0.00%)	0(0.00%)	0(0.00%)	0(0.00%)	0(0.00%)
Unknown/other	263446(43.78%)	104091(42.20%)	159355(44.88%)	42781(46.36%)	220665(43.32%)	0(0.00%)	0(0.00%)	0(0.00%)	0(0.00%)	0(0.00%)
Cancer	104766(17.41%)	45152(18.30%)	59614(16.79%)	14872(16.12%)	89894(17.65%)	553743(9.79%)	190338(11.57%)	363405(9.05%)	100403(9.24%)	453340(9.92%)
Depression	85945(14.28%)	44035(17.85%)	41910(11.80%)	14150(15.33%)	71795(14.09%)	368371(6.51%)	164776(10.01%)	203595(5.07%)	82464(7.59%)	285907(6.25%)
Back pain	149061(24.77%)	85033(34.47%)	64028(18.03%)	31778(34.44%)	117283(23.02%)	1090779(19.28%)	502619(30.55%)	588160(14.66%)	310588(28.58%)	780191(17.07%)
Neck pain	40434(6.72%)	22554(9.14%)	17880(5.04%)	9656(10.46%)	30778(6.04%)	341286(6.03%)	147259(8.95%)	194027(4.83%)	100441(9.24%)	240845(5.27%)
Arthritis	418400(69.53%)	192896(78.20%)	225504(63.51%)	72236(78.28%)	346164(67.95%)	2944626(52.04%)	1095780(66.60%)	1848846(46.07%)	722072(66.44%)	2222554(48.61%)
Headache	4423(0.74%)	2775(1.12%)	1648(0.46%)	1136(1.23%)	3287(0.65%)	35964(0.64%)	18489(1.12%)	17475(0.44%)	11528(1.06%)	24436(0.53%)
HIV	1697(0.28%)	822(0.33%)	875(0.25%)	325(0.35%)	1372(0.27%)	6961(0.12%)	2764(0.17%)	4197(0.10%)	1704(0.16%)	5257(0.11%)

Abbreviations: Chronic kidney disease (CKD), hypertension (HTN), cardiovascular disease (CVD), diabetes (DM) and human immunodeficiency virus (HIV)
Significant group differences were observed in all of characteristics, assessed by t-test and chi-square test (p<0.0001)

© 2020 Han Y et al. *JAMA Network Open*.

eFigure 3. Trends in total annual days' supply of opioids and prescription NSAIDs by CKD status, the yearly cohort 2006-2015

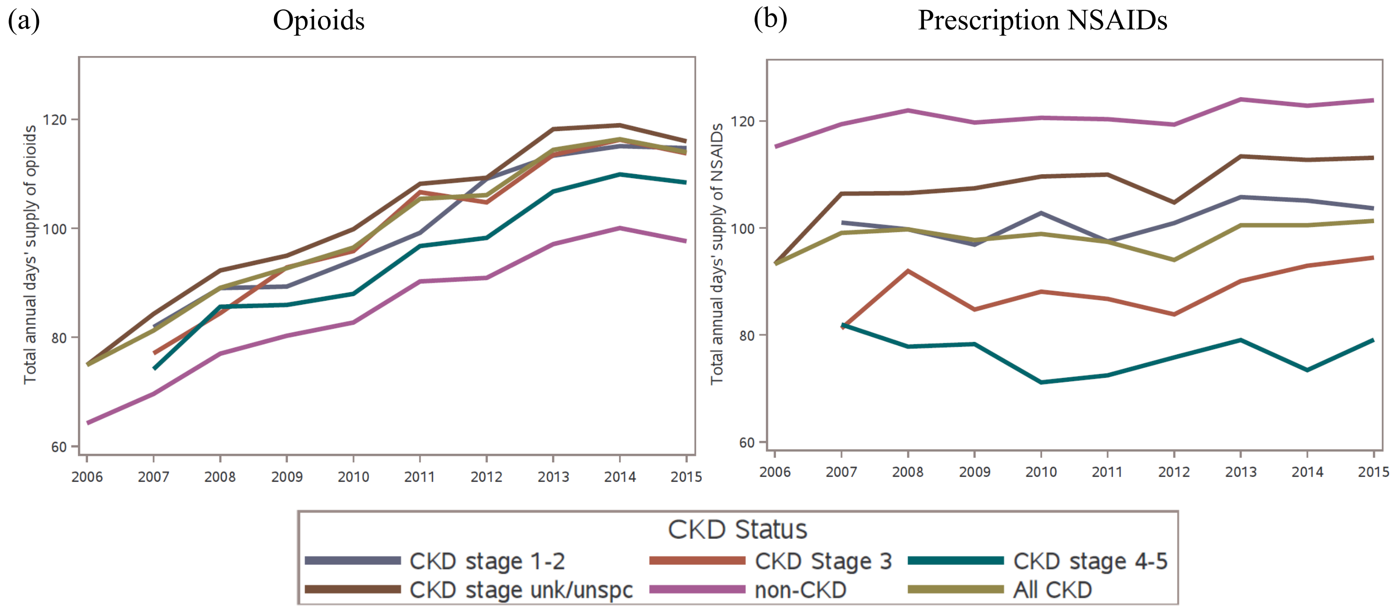

(a) The average number of days/year of opioid analgesics prescribed among users rose from 75 days/ year in 2006 to 114 days/year in 2014, with a slight decline after 2014. (b) The average days of NSAIDs prescribed per user remained stable during 2006-2015, ranging from 93-101 days per person.

© 2020 Han Y et al. *JAMA Network Open*.

eFigure 4. Trends in proportion of prescribed opioids and NSAIDs by CKD and race, the yearly cohort 2006-2015

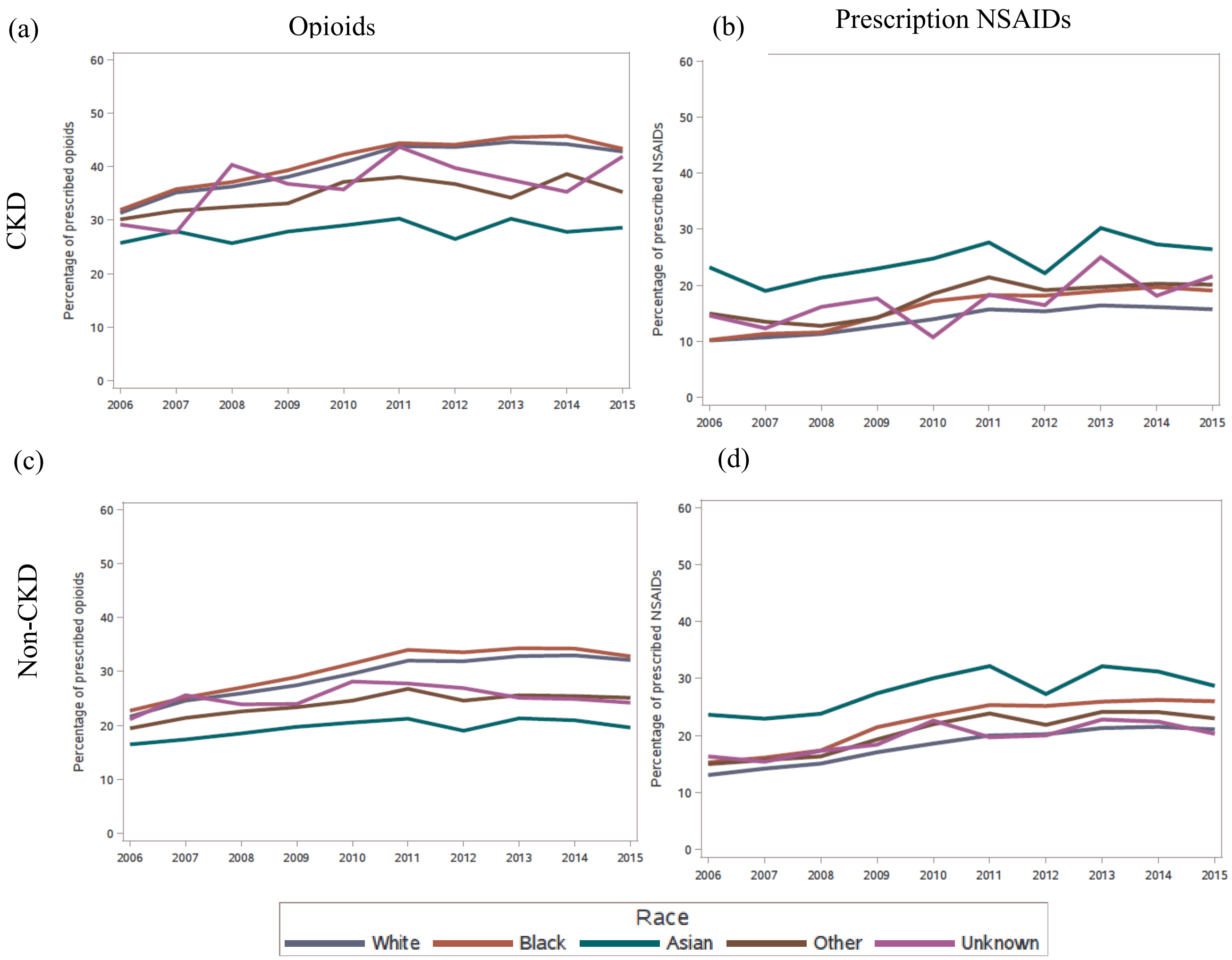

© 2020 Han Y et al. *JAMA Network Open*.

eFigure 5. Trends in use of opioids and prescription NSAIDs by CKD and selected comorbidities, the yearly cohort 2006-2015*

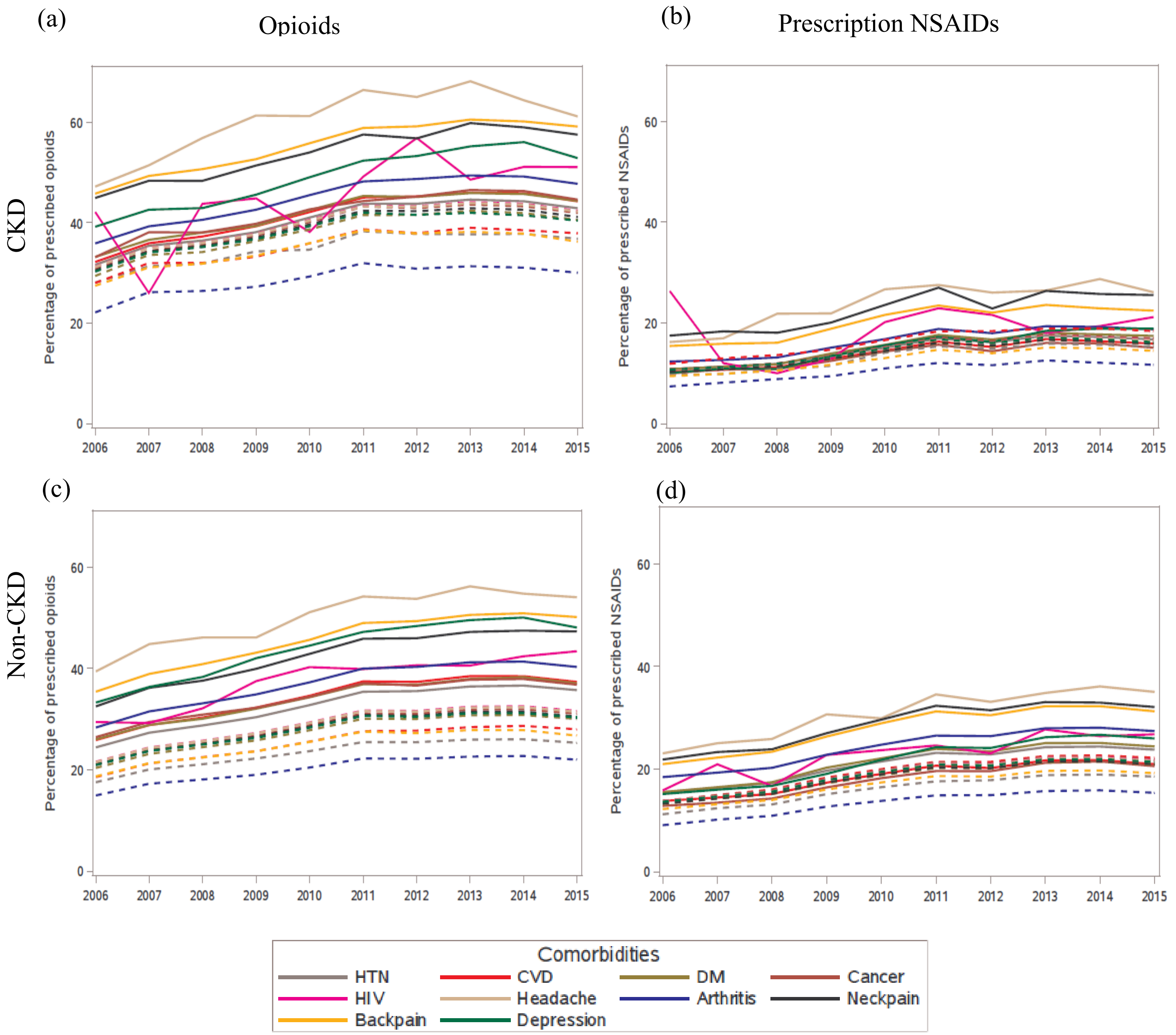

*Solid line represented patients with the specified disease, and dash lines represented those without the specified disease.
Abbreviations: hypertension (HTN), cardiovascular disease (CVD), diabetes (DM) and human immunodeficiency virus (HIV)

© 2020 Han Y et al. *JAMA Network Open*.

eTable 4. Results of trend analyses for opioid and prescription NSAID use according to CKD status, the yearly cohort 2006-2015

Measures	Time	Non-CKD	CKD stage1-2	CKD stage3	CKD stage4-5	CKD stage unknown/other
Ever used opioids						
Model1	1.06, (1.06,1.07)					
Model2	1.06, (1.06,1.06)	ref	1.27, (1.25,1.30)	1.33, (1.32,1.34)	1.43, (1.41,1.45)	1.26, (1.25,1.28)
Model3	1.06, (1.06,1.06)	ref	1.44, (1.37,1.52)	1.53, (1.48,1.57)	1.63, (1.56,1.71)	1.37, (1.34,1.40)
Model4	1.07, (1.06,1.07)	ref	1.46, (1.38,1.53)	1.55, (1.50,1.59)	1.67, (1.60,1.75)	1.38, (1.34,1.41)
Prescribed opioids >90 days						
Model1	1.12, (1.11,1.12)					
Model2	1.12, (1.11,1.12)	ref	1.08, (1.03,1.12)	1.07, (1.05,1.10)	1.05, (1.00,1.09)	1.08, (1.06,1.10)
Model3	1.12, (1.12,1.12)	ref	1.24, (1.08,1.43)	1.27, (1.17,1.37)	1.30, (1.13,1.49)	1.23, (1.16,1.32)
Model4	1.12, (1.11,1.12)	ref	1.24, (1.07,1.43)	1.26, (1.16,1.36)	1.29, (1.12,1.47)	1.22, (1.15,1.31)
Ever used prescription NSAIDs						
Model1	1.04, (1.04,1.05)					
Model2	1.05, (1.05,1.05)	ref	0.80, (0.78,0.82)	0.67, (0.66,0.68)	0.52, (0.50,0.53)	0.82, (0.81,0.83)
Model3	1.05, (1.05,1.05)	ref	0.90, (0.84,0.97)	0.87, (0.83,0.90)	0.68, (0.64,0.73)	0.92, (0.89,0.94)
Model4	1.06, (1.06,1.06)	ref	0.92, (0.86,0.99)	0.88, (0.84,0.92)	0.70, (0.65,0.75)	0.95, (0.92,0.98)
Prescribed NSAIDs >90 days						
Model1	1.02, (1.02,1.02)					
Model2	1.02, (1.02,1.03)	ref	0.86, (0.81,0.91)	0.68, (0.66,0.71)	0.61, (0.56,0.67)	0.88, (0.85,0.90)
Model3	1.02, (1.02,1.03)	ref	1.07, (0.89,1.28)	0.84, (0.74,0.95)	0.97, (0.77,1.23)	1.03, (0.95,1.12)
Model4	1.02, (1.02,1.03)	ref	1.08, (0.89,1.31)	0.84, (0.73,0.96)	0.91, (0.71,1.17)	1.04, (0.96,1.13)
Note: Odds ratio (95% confidence intervals) in opioids and prescription NSAIDs use, compared to non-CKD population as reference. Model 1 adjusted for time; Model 2 adjusted for time and CKD status; Model 3 adjusted for time, CKD status, and interaction CKD status×time. Model 4 adjusted for time, CKD status, interaction CKD status×time, age, race and gender. P value for interaction CKD status×time <0.05 except for Model 3 and Model 4 in prescribed opioids >90 days						
Abbreviations: Chronic kidney disease (CKD)						

© 2020 Han Y et al. *JAMA Network Open*.

eFigure 6. Changes in proportion of prescribed opioids among CKD and without CKD from year 2006-2015*

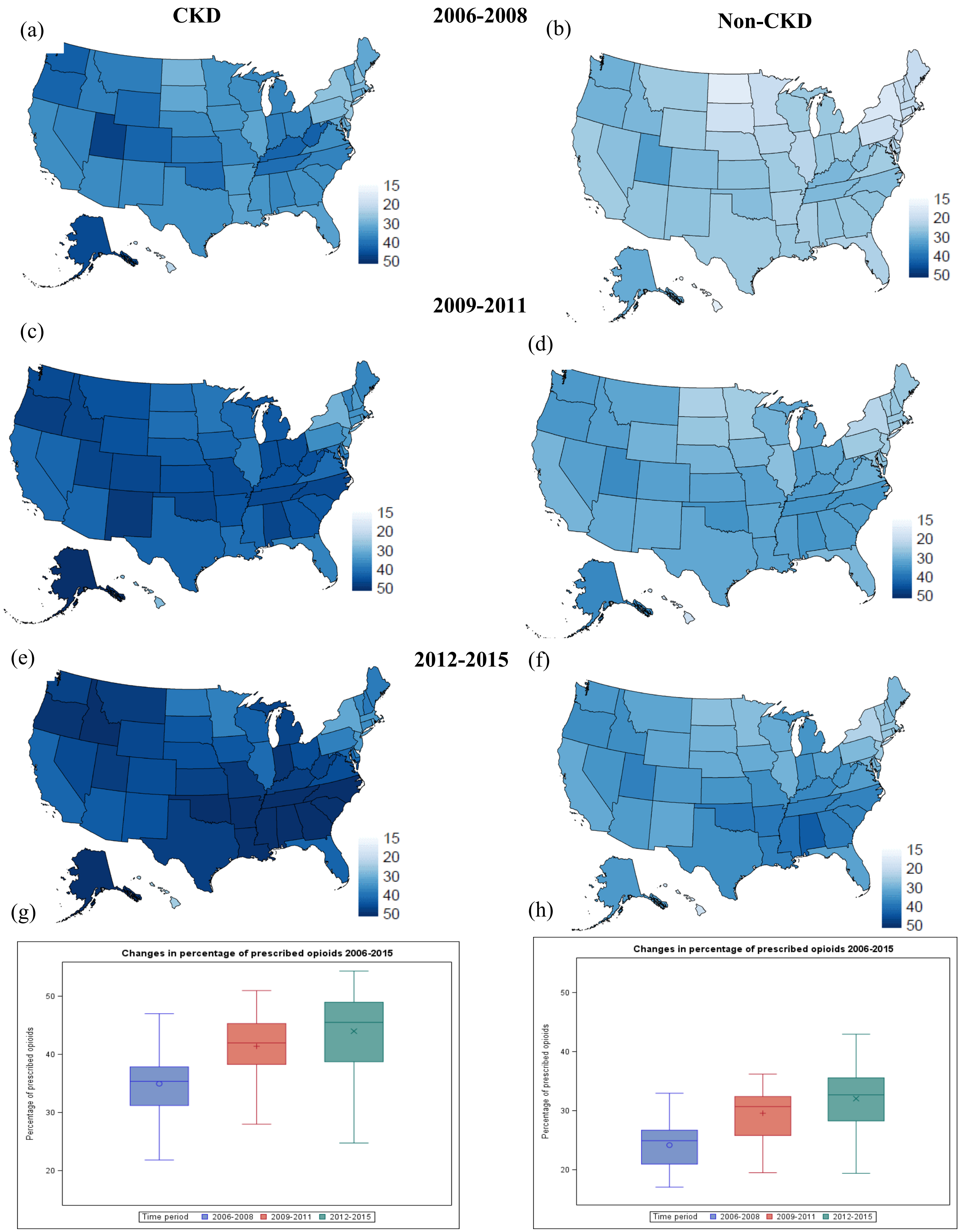

*Value below 15 and above 50 were coded as 15 and 50

© 2020 Han Y et al. *JAMA Network Open*.

eFigure 7. Changes in proportion of prescribed NSAIDs among CKD and without CKD from year 2006-2015*

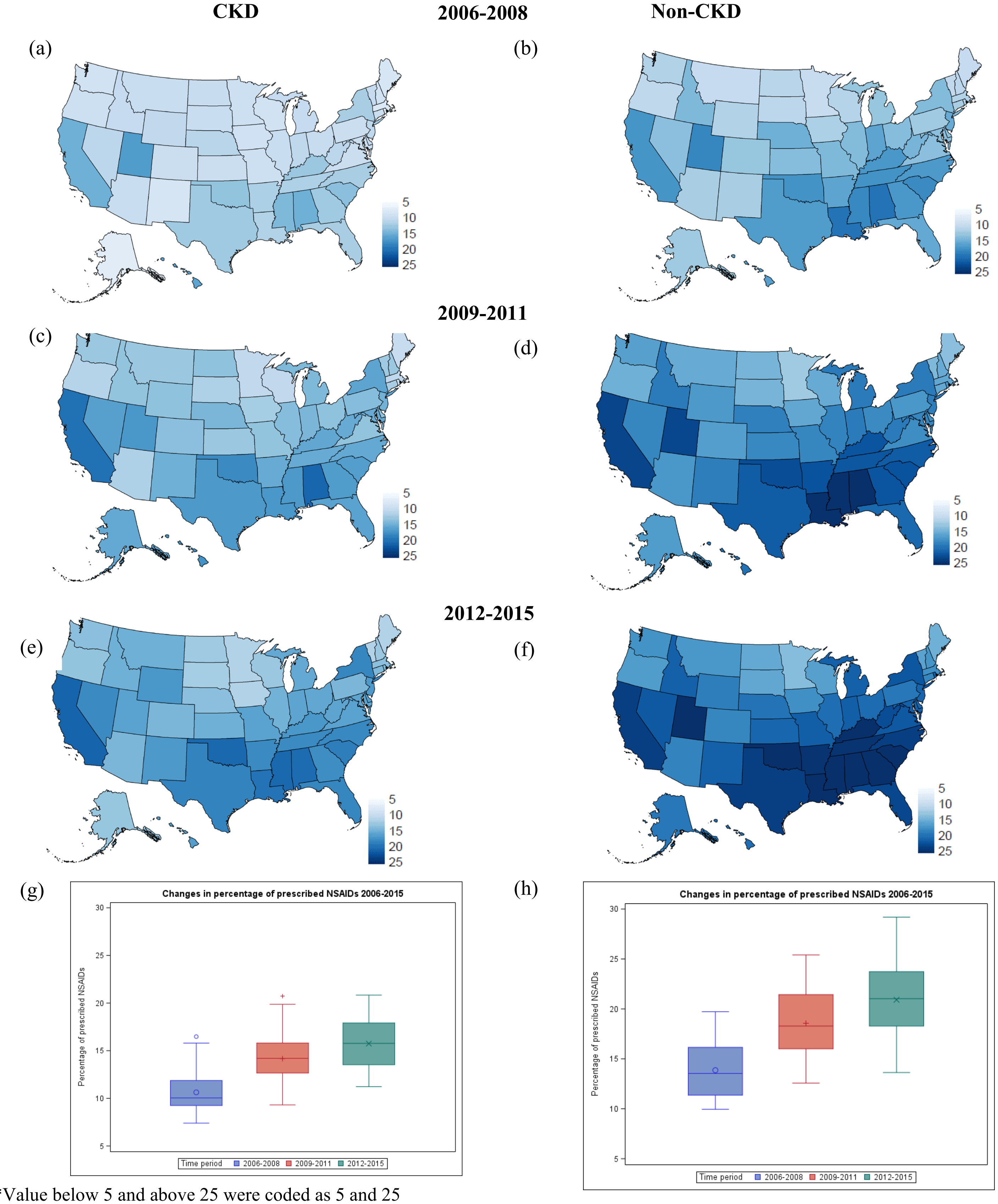

*Value below 5 and above 25 were coded as 5 and 25

© 2020 Han Y et al. *JAMA Network Open*.

eFigure 8. Changes in proportion of prescribed opioids greater than 90 days among users with CKD and without CKD from 2006-2015*

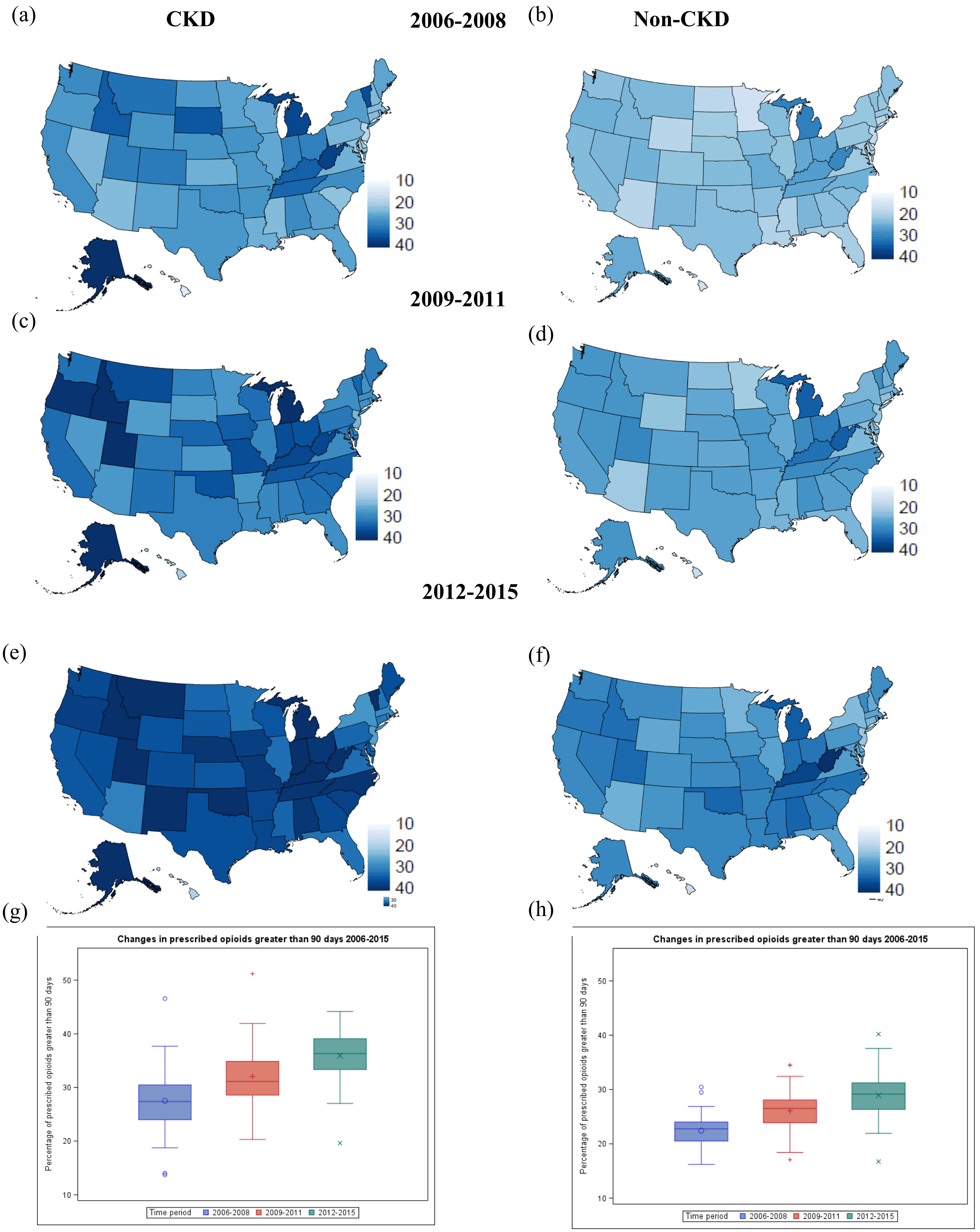

*Value below 10 and above 40 were coded as 10 and 40

© 2020 Han Y et al. *JAMA Network Open*.

eFigure 9. Changes in proportion of prescribed NSAIDs greater than 90 days among users with CKD and without CKD from 2006-2015*

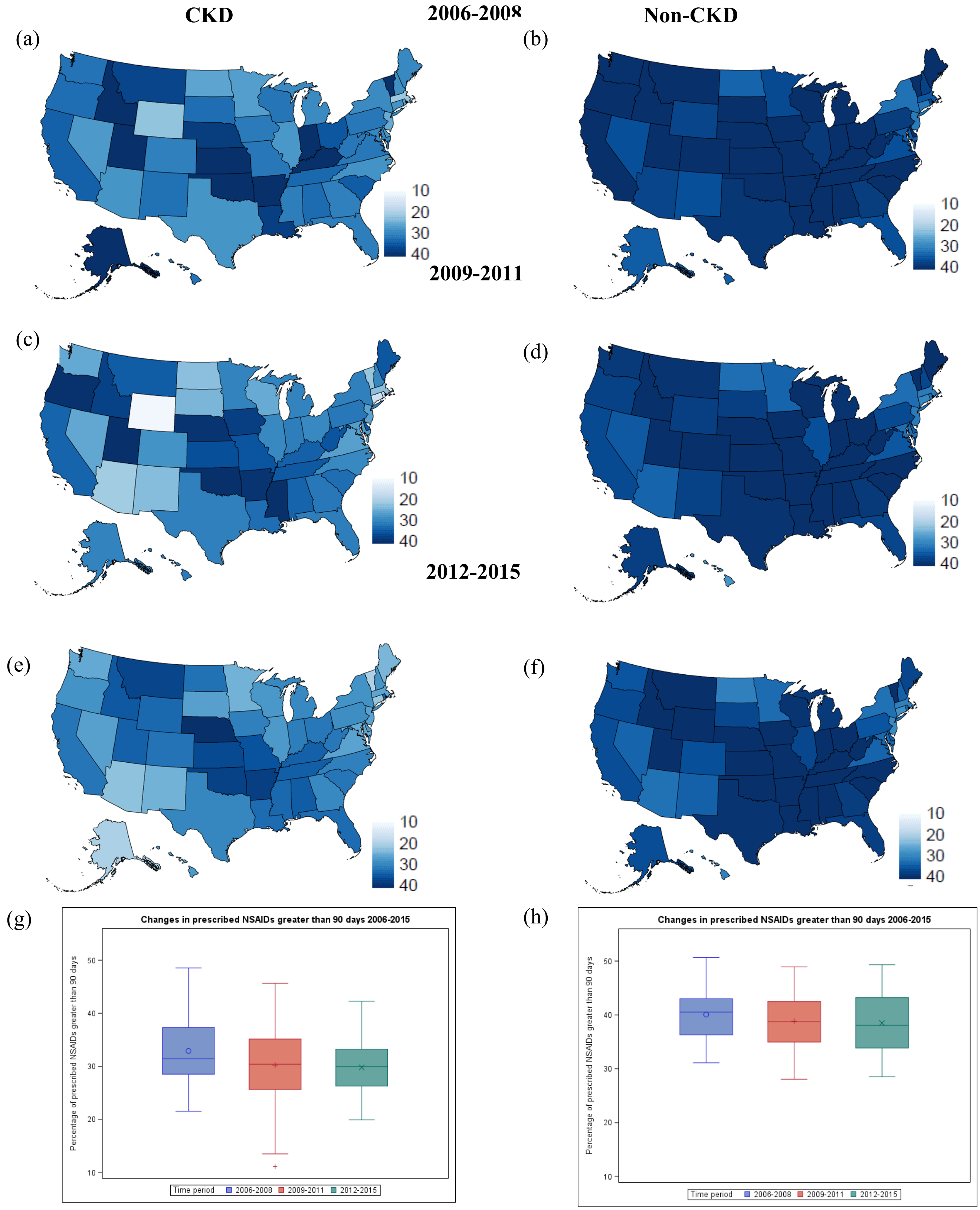

*Value below 10 and above 40 were coded as 10 and 40

© 2020 Han Y et al. *JAMA Network Open*.

eFigure 10. Changes in total annual days' supply of opioids among users with CKD and without CKD from 2006-2015*

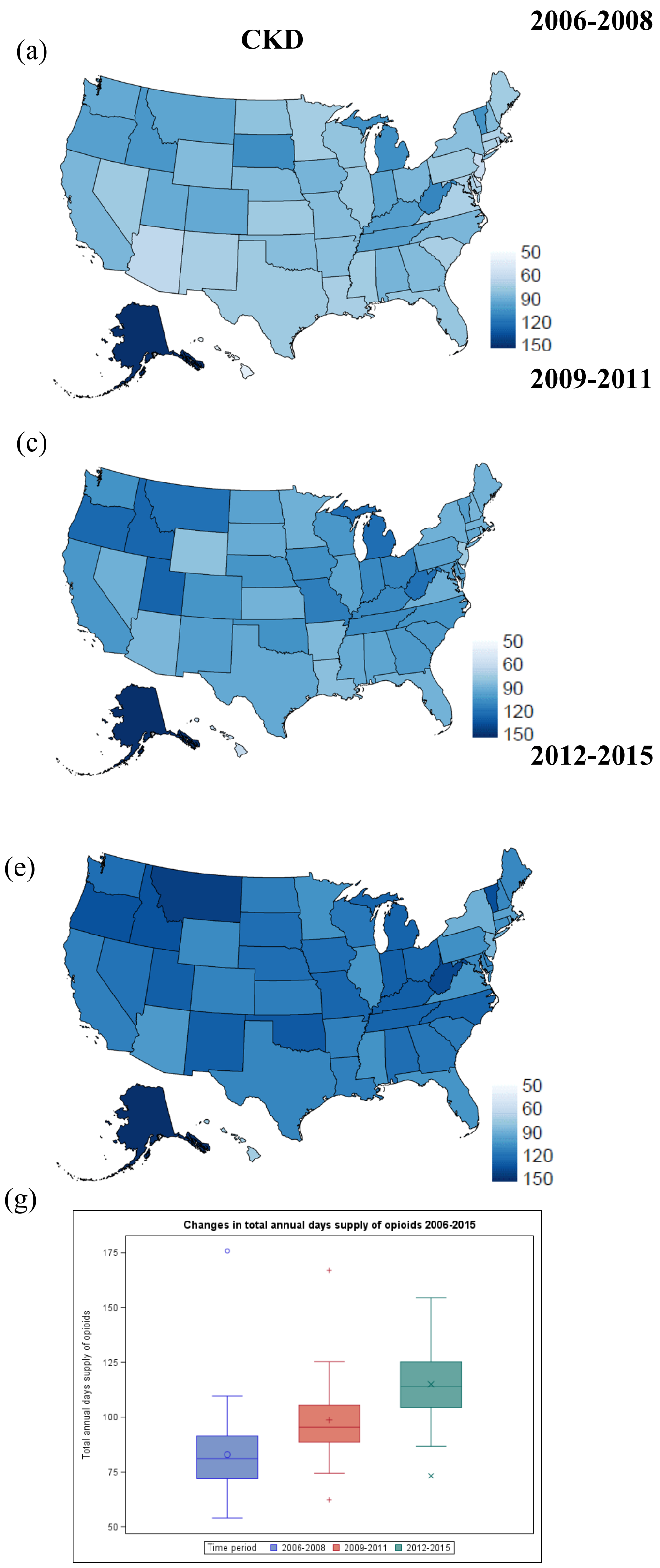

*Value below 50 and above 150 were coded as 50 and 150

© 2020 Han Y et al. *JAMA Network Open*.

eFigure 11. Changes in total annual days' supply of prescription NSAIDs among users with CKD and without CKD from 2006-2015*

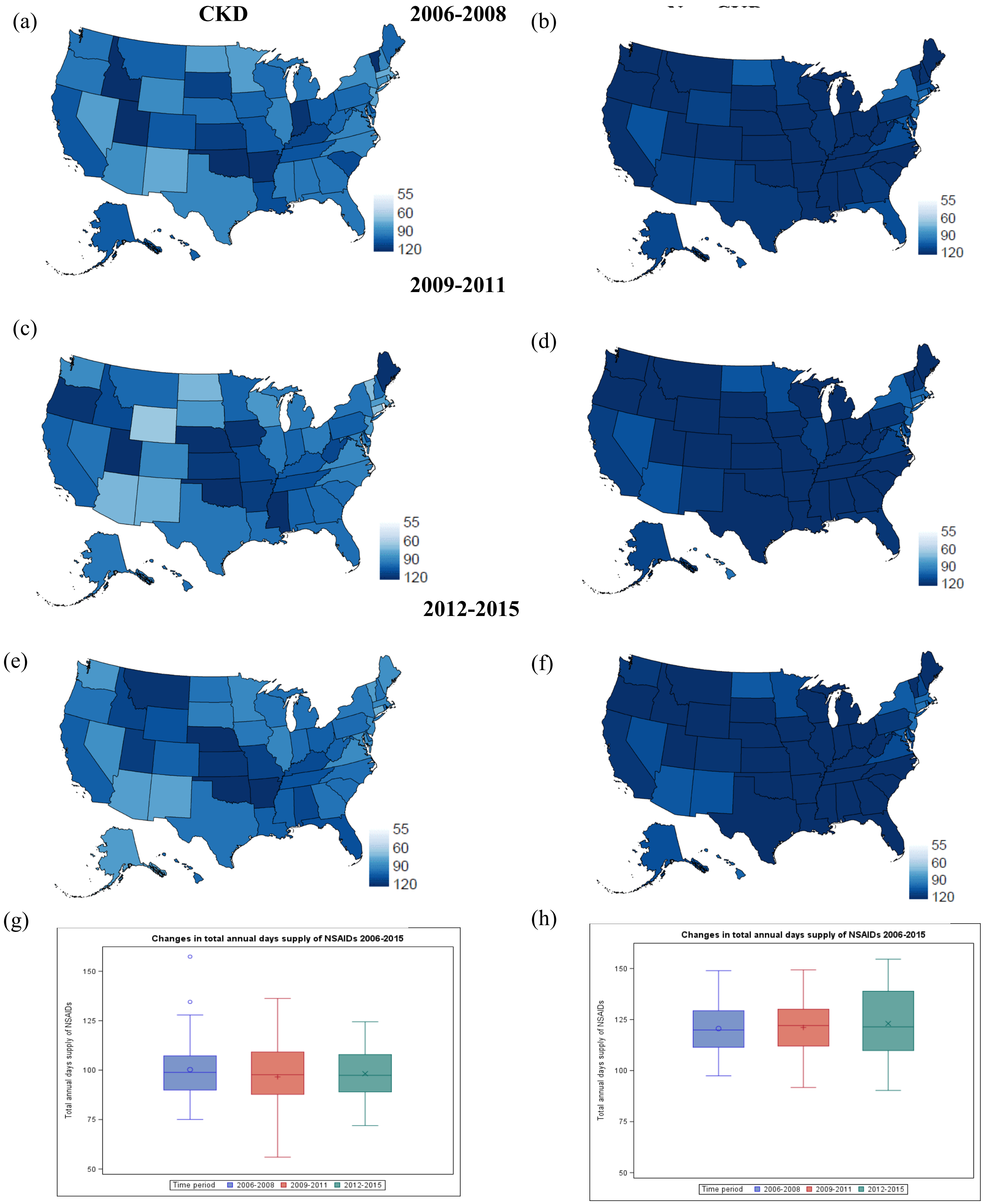

*Value below 55 and above 120 were coded as 55 and 120

© 2020 Han Y et al. *JAMA Network Open*.

eTable 5. Results of generalized linear models for the relationship of patients' baseline characteristics to opioid and prescription NSAID use (as prescribed greater than 90 days), the yearly cohort 2006-2015

	Opioids >90 days			Prescription NSAIDs >90 days		
**Characteristics**	**OR**	**95%CI**	**P value**	**OR**	**95%CI**	**P value**
Age group						
65-75	ref	ref	ref	ref	ref	ref
75-85	1.36	1.34-1.38	<.0001	1.05	1.03-1.06	<.0001
85 and above	1.80	1.76-1.83	<.0001	1.11	1.09-1.14	<.0001
Gender						
Male	ref	ref	ref	ref	ref	ref
Female	1.32	1.30-1.33	<.0001	1.20	1.19-1.22	<.0001
Race						
White	ref	ref	ref	ref	ref	ref
Asian	0.52	0.49-0.55	<.0001	1.07	1.04-1.10	<.0001
Other	0.92	0.88-0.97	0.0006	0.87	0.83-0.90	<.0001
Black	1.17	1.15-1.20	<.0001	0.96	0.94-0.98	0.0001
Unknown	1.01	0.92-1.10	0.8947	1.22	1.13-1.33	<.0001
HTN	1.08	1.07-1.10	<.0001	1.04	1.03-1.06	<.0001
CVD	1.09	1.08-1.10	<.0001	0.95	0.94-0.96	<.0001
DM	1.16	1.14-1.18	<.0001	1.07	1.05-1.08	<.0001
CKD status						
non-CKD	ref	ref	ref	ref	ref	ref
CKD stage 1-2	1.15	1.11-1.19	<.0001	0.86	0.81-0.91	<.0001
CKD stage 3	1.19	1.16-1.21	<.0001	0.68	0.65-0.70	<.0001
CKD stage 4-5	1.16	1.12-1.21	<.0001	0.59	0.54-0.64	<.0001
Unknown/other	1.08	1.06-1.10	<.0001	0.86	0.84-0.89	<.0001
Cancer	1.09	1.07-1.10	<.0001	0.93	0.92-0.95	<.0001
Depression	1.10	1.08-1.11	<.0001	1.04	1.02-1.06	<.0001
Back pain	1.26	1.24-1.27	<.0001	1.08	1.07-1.09	<.0001
Neck pain	1.03	1.02-1.05	<.0001	1.02	1.00-1.04	0.0137
Arthritis	1.20	1.19-1.22	<.0001	1.20	1.18-1.21	<.0001
Headache	1.04	1.00-1.09	0.0495	0.99	0.94-1.04	0.6516
HIV	1.15	1.04-1.28	0.0062	0.86	0.74-1.01	0.0591
Abbreviations: Chronic kidney disease (CKD), hypertension (HTN), cardiovascular disease (CVD), diabetes (DM) and human immunodeficiency virus (HIV)						

© 2020 Han Y et al. *JAMA Network Open*.

eTable 6. Results of multivariable-adjusted Cox models for the association of opioid and prescription NSAID use (as total annual days' supply) with CKD outcomes

	ESRD*			All-cause mortality		
**Characteristics**	**HR**	**95%CI**	**P value**	**HR**	**95%CI**	**P value**
Total annual days' supply of opioids						
0 days	ref	ref	ref	ref	ref	ref
1-90 days	1.07	1.00-1.14	0.0393	1.07	1.06-1.08	<.0001
91-180 days	1.26	1.11-1.44	0.0004	1.42	1.39-1.45	<.0001
>180 days	1.17	1.05-1.31	0.0057	1.70	1.68-1.73	<.0001
Total annual days' supply of NSAIDs						
0 days	ref	ref	ref	ref	ref	ref
1-90 days	0.98	0.91-1.06	0.6322	0.81	0.80-0.82	<.0001
91-180 days	0.77	0.65-0.91	0.0018	0.82	0.80-0.84	<.0001
>180 days	0.97	0.85-1.11	0.6324	0.92	0.90-0.94	<.0001
Age group						
65-75	0.83	0.78-0.87	<.0001	2.14	2.12-2.16	<.0001
75-85	0.43	0.39-0.49	<.0001	5.22	5.17-5.28	<.0001
85 and above						
Gender						
Male	ref	ref	ref	ref	ref	ref
Female	0.75	0.71-0.79	<.0001	0.79	0.78-0.80	<.0001
Race						
White	ref	ref	ref	ref	ref	ref
Asian	1.69	1.51-1.90	<.0001	0.66	0.64-0.67	<.0001
Other	1.49	1.27-1.74	<.0001	0.84	0.82-0.87	<.0001
Black	2.08	1.95-2.22	<.0001	1.06	1.05-1.07	<.0001
Unknown	0.83	0.45-1.55	0.5619	0.92	0.85-1.00	0.0559
HTN	1.50	1.38-1.62	<.0001	0.97	0.96-0.98	<.0001
CVD	1.31	1.24-1.40	<.0001	1.63	1.62-1.65	<.0001
DM	2.24	2.11-2.37	<.0001	1.25	1.24-1.26	<.0001
CKD status						
non-CKD	ref	ref	ref	ref	ref	ref
CKD stage 1-2	6.56	5.41-7.94	<.0001	1.35	1.29-1.41	<.0001
CKD stage 3	10.78	9.85-11.80	<.0001	1.41	1.37-1.44	<.0001
CKD stage 4-5	51.02	47.47-54.83	<.0001	2.04	1.98-2.09	<.0001
Unknown/other	5.68	5.22-6.18	<.0001	1.55	1.53-1.57	<.0001
Cancer	1.01	0.93-1.10	0.7678	1.31	1.29-1.33	<.0001
Depression	0.81	0.71-0.91	0.0005	1.63	1.61-1.65	<.0001
Back pain	0.87	0.81-0.94	0.0006	0.83	0.82-0.84	<.0001
Neck pain	0.92	0.80-1.04	0.1864	0.82	0.80-0.83	<.0001
Arthritis	0.82	0.78-0.87	<.0001	1.11	1.10-1.12	<.0001

© 2020 Han Y et al. *JAMA Network Open*.

Headache	0.72	0.47-1.10	0.1292	0.72	0.67-0.76	<.0001
HIV	1.33	0.73-2.40	0.3494	1.64	1.45-1.85	<.0001
* A competing risk model (accounting for death) was used to estimate the hazard ratio (HR) for development of ESRD						
Abbreviations: Chronic kidney disease (CKD), hypertension (HTN), cardiovascular disease (CVD), diabetes (DM) and human immunodeficiency virus (HIV)						

© 2020 Han Y et al. *JAMA Network Open*.
